# Short Activators and Repressors of RNA Toehold Switches

**DOI:** 10.1021/acssynbio.2c00641

**Published:** 2023-02-21

**Authors:** Megan A. McSweeney, Yan Zhang, Mark P. Styczynski

**Affiliations:** School of Chemical & Biomolecular Engineering, Georgia Institute of Technology, Atlanta, Georgia 30332, United States

**Keywords:** toehold switch, biosensor, cell-free expression, microRNA

## Abstract

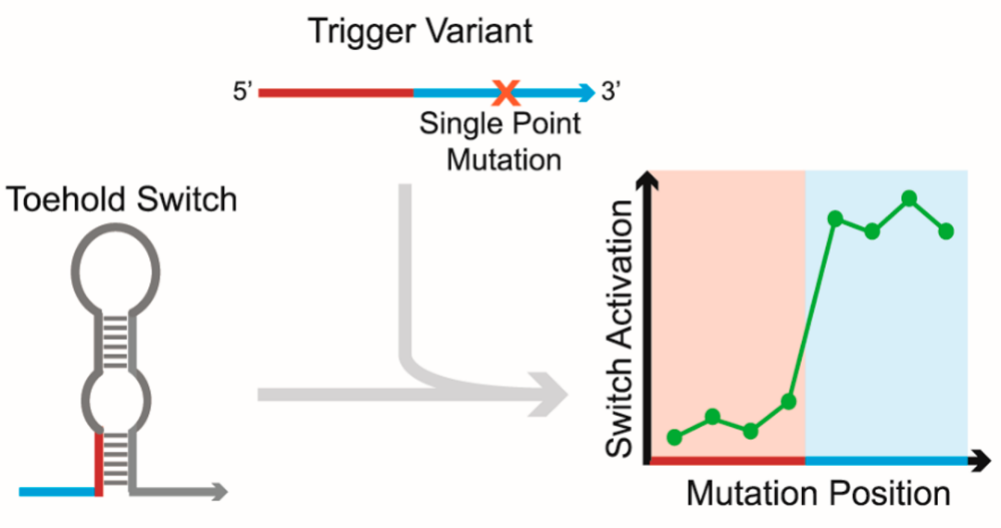

RNA toehold switches are a widely used class of molecule to detect specific RNA “trigger” sequences, but their design, intended function, and characterization to date leave it unclear whether they can function properly with triggers shorter than 36 nucleotides. Here, we explore the feasibility of using standard toehold switches with 23-nucleotide truncated triggers. We assess the crosstalk of different triggers with significant homology and identify a highly sensitive trigger region where just one mutation from the consensus trigger sequence can reduce switch activation by 98.6%. However, we also find that triggers with as many as seven mutations outside of this region can still lead to 5-fold induction of the switch. We also present a new approach using 18- to 22-nucleotide triggers as translational repressors for toehold switches and assess the off-target regulation for this strategy as well. The development and characterization of these strategies could help enable applications like microRNA sensors, where well-characterized crosstalk between sensors and detection of short target sequences are critical.

## Introduction

Biosensors that use cell-free expression (CFE) systems have immense promise as low-cost disease diagnostics for use at the point of care. CFE biosensors have been developed to sense and respond to many classes of biomarkers,^1^ including small molecules,^2,3^ ions,^4^ and nucleic acids.^5,6^ Many CFE biosensors engineered to detect nucleic acid biomarkers, such as pathogenic RNA, use RNA toehold switches to regulate signal output.^1^ Toehold switches are *de novo* designed riboregulators that can sensitively and specifically detect arbitrary RNA target sequences referred to as “triggers.”^7^ Toehold switches have been successfully implemented in paper-based biosensors for Ebola,^6^ Zika,^8^ and SARS-CoV-2^9,10^ RNA biomarker detection.

Since toehold switches were first reported, there have been two principal designs used for their implementation. The original “Series A” toehold switch design employed a 62-nt RNA trigger (not including the transcriptional terminator and nonbinding GGG at the 5′ end).^7^ The most recent and more optimized “Series B” toehold switch design uses a shorter 36-nt RNA trigger^8^ (Figure S1). On the basis of the reported characterization of these toehold switch designs to date, it was unclear whether they could work with RNA triggers shorter than 36 nt; the original characterization of the Series A design reported success with triggers as short as 54 nt,^7^ and to our knowledge the Series B design has not been tested with triggers shorter than 36 nt. Pushing the limits of switch activation with truncated triggers would be critical for applications where the sensing of short RNA oligonucleotides (“oligos”) is desired.

One potentially relevant and impactful application for sensing short RNA oligos would be microRNA (miRNA) detection. miRNAs are short (18–24 nt),^11,12^ endogenous, noncoding RNA sequences present in numerous bodily fluids^13^ and cell lines^14^ with an important role in gene regulation. miRNA sequences have been identified as potentially useful biomarkers indicative of many conditions, including heart disease,^15^ kidney disease,^16^ and many cancers.^17−19^ miRNA measurement typically relies on miRNA expression profiling technologies, such as microarrays, reverse-transcription quantitative polymerase chain reaction (RT-qPCR), and next generation sequencing.^20^ RT-qPCR remains the gold standard for miRNA identification because of its high specificity and sensitivity, but suffers from being relatively expensive and low-throughput.^20^

One recent report suggests that using toehold switches for miRNA measurement is, in fact, feasible,^21^ though there remains some ambiguity as to whether these approaches can be implemented in CFE systems for low-cost diagnostic biosensors. Two toehold switch sensors for two different miRNA were implemented *in vivo* in mammalian cells, though by using a unique switch design schema rather than the more widely used Series A or B designs. Upon activation, these *in vivo* switches yielded an approximately 2-fold increase in signal, which is an output orders of magnitude lower than for Series A or B designs and potentially challenging to use reliably for diagnostic or research applications. We hypothesized that if Series B toehold switch designs—which were developed to minimize leak and, thus, increase signal-to-noise levels—were used with truncated miRNA-length triggers, these sensors could retain functionality and may even have better performance characteristics than the recently reported *in vivo* sensors.

Here, we show that Series B toehold switches can be used to reliably detect 22- and 23-nt triggers without altering the switch sequence design. We characterize the functionality of toehold switches with these truncated triggers by investigating the position-specific impact of mutations from the consensus target sequence on switch output. To further broaden the potential sensing applications, we show that with properly designed switches, truncated target sequences can be used as toehold switch repressors that are less sensitive to mutations from the consensus target sequence than widely used toehold switch activators. These characterizations of switch specificity for different trigger sequences are critical for the implementation of toehold switches for miRNA sensing applications since miRNA targets can have significant sequence similarity to each other.

## Results and Discussion

First, we demonstrated that the Series B toehold switch design is compatible with triggers shorter than 36 nt. In previous work using one Series A switch, truncated triggers generated by removing nucleotides from either the 3′ or 5′ end of the consensus trigger still activated the toehold switch, but this robustness was only demonstrated for shortening the trigger from 62 nt to 54 nt. A different Series A toehold switch had a surprisingly strong response from triggers as short as 13 nt after removing bases from the 5′ end, but this behavior was not expected to be generalizable to all Series A toehold switches. Series B switches have more desirable performance characteristics for sensing applications,^8^ yet the robustness of Series B switches to truncated triggers has not previously been investigated. We tested two different Series B toehold switches for activation in CFE with truncated complementary triggers either 22 or 23 nt in length. Both truncated triggers induced strong responses from their cognate switches (Figure S2), thereby demonstrating that the established Series B toehold switch design can function with triggers as short as 22 nt.

We next sought to assess the degree of specificity of some toehold switches for truncated triggers. For most diagnostic applications, sensing mechanisms with high orthogonality and little crosstalk—that is, little response of one sensor to the target of another sensor—are desirable for avoiding false positives. Other diagnostic applications might benefit from sensing mechanisms that have lower specificity—for example, a sensor able to detect two different strain variants of the same virus. Accordingly, an assessment of the expected specificity of these switches when using truncated triggers is critical for determining their suitability for downstream applications.

**Figure 1:**
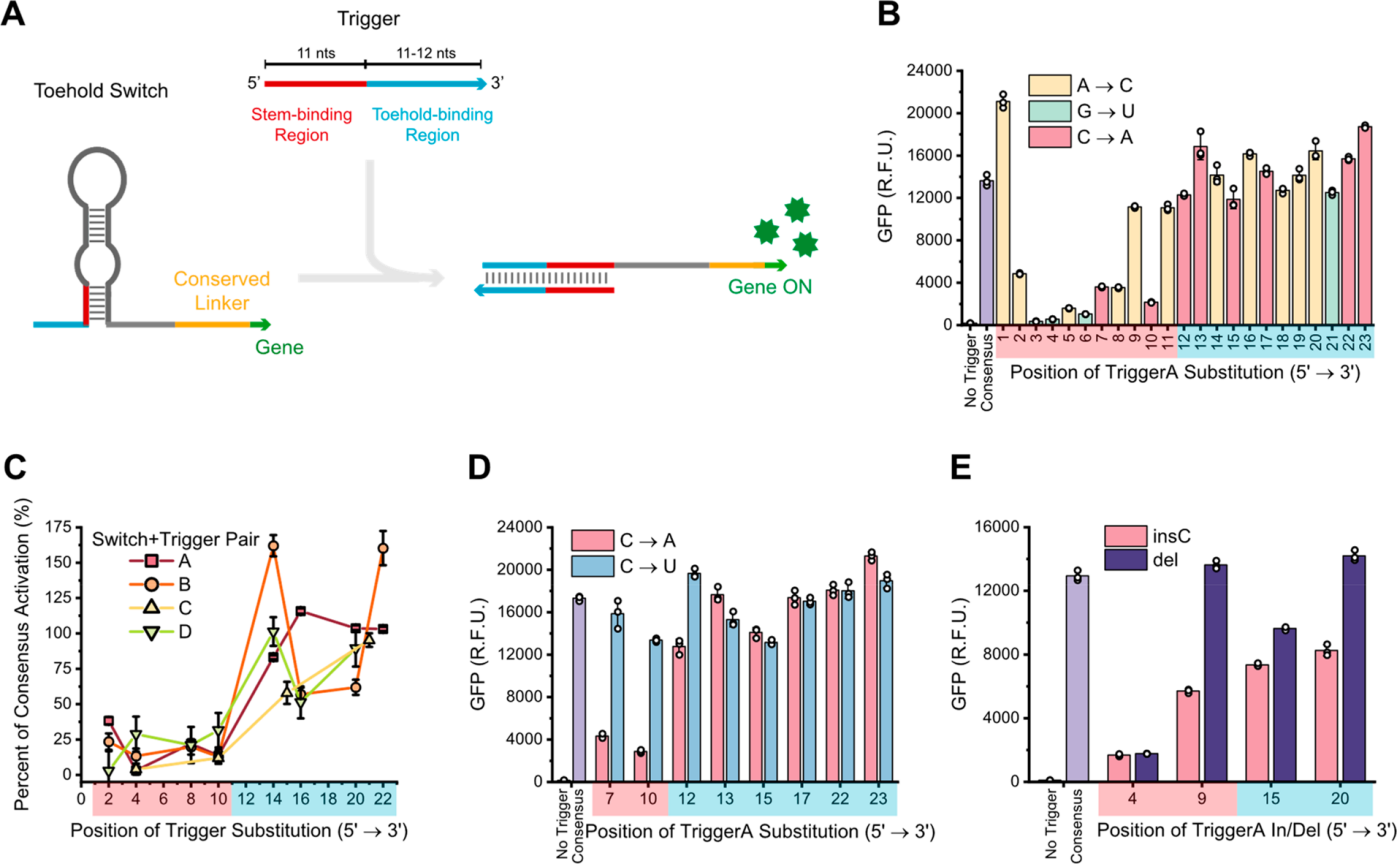
Impacts of trigger point mutations on switch activation are dependent on their position in the trigger. (A) Schematic of toehold switch activation with truncated triggers. The trigger cartoon identifies the location of the stem-binding (red) and toehold-binding (blue) regions. (B) Effects of single point mutations at every position in TriggerA on SwitchA activation. (C) Generalizability of a mutation-sensitive stem-binding region and mutation-robust toehold-binding region across four different switch and trigger pairs. Triggers A and B are each 23 nt, while Triggers C and D are 22 nt. (D) SwitchA robustness to wobble mutation at every position in TriggerA. (E) The impacts of insertion and deletion trigger mutations on switch performance. The *x* axes are shaded to indicate positions within the stem-binding region (red) or toehold-binding region (blue). Error bars in panels B, D, and E represent the standard deviation of technical triplicates, with white circles indicating individual measurements. Error bars in panel C represent standard deviation of the percent of consensus activation, as calculated by propagating the error from raw GFP measurements.

To assess *in vitro* switch specificity for truncated triggers, trigger variants with single point mutations compared with the switch’s cognate complementary sequence were synthesized and characterized in CFE with a green fluorescent protein (GFP) reporter. Figure 1A shows a schematic of toehold switch activation via truncated triggers, with the trigger’s stem-binding and toehold-binding regions explicitly identified. We first tested only a specific set of transversions to facilitate comparison of the effects of mutations across different trigger positions and to avoid potential confounding effects of wobble base pairing. We found that activation of SwitchA was highly robust to substitutions made in the toehold-binding region at the 3′ end of its cognate-truncated trigger (TriggerA), while highly sensitive to substitutions made in the stem-binding region at the 5′ end of TriggerA (Figure 1B). Notably, a TriggerA variant with one substitution at position 3 (counting from the 5′ end) resulted in a 98.6% decrease in SwitchA activation compared with the consensus TriggerA. Multiple TriggerA variants unexpectedly increased activation compared with consensus TriggerA. The significant (Table S1) and largely consistent difference in mutation impact on switch activation between the stem-binding and toehold-binding regions suggests that mutation impacts are strongly influenced by the secondary structure of the switch.

The trend of activation sensitivity to mutations in the stem-binding region and increased robustness to mutations in the toehold region is generally conserved across multiple unique switch and trigger pairs, with minor switch-specific differences (Figures 1C and S3), which further suggests that this phenomenon is not switch- or sequence-specific but instead related to RNA secondary structures. All four of the tested switches showed high sensitivity to triggers with mutations in the first 10 nucleotides, despite variation in the strength of the toehold switches and the lengths of the triggers (Figure 1C). Though the impacts of mutations in the toehold-binding region of the triggers had higher variability across switches, all yielded significantly higher switch activation than mutations in the stem-binding region.

We then tested triggers with substitutions that changed a canonical Watson–Crick base pair to a G-U wobble base pair to see if the position-specific trend was consistent for different types of substitutions. Activation of SwitchA by TriggerA variants, each with a single C mutated to a U, was compared with activation by variants where that C was mutated to an A. In contrast to nonwobble substitutions, triggers with wobble substitutions were generally robust even when the mutation was in the stem-binding region; these triggers performed similarly to the consensus trigger (Figure 1D). As a result, the difference in SwitchA activation between a C-A mutated trigger and a C-U mutated trigger was greatest when that mutation was in the stem-binding region of the trigger, while the difference was much smaller—and sometimes negligible—when these mutations were in the toehold-binding region. However, this trend was not consistently observed in SwitchB, where two positions in the stem-binding region showed the same activation for triggers with wobble and nonwobble substitutions, but one position showed different activation (Figure S4). This indicates that the relative impact of wobble versus nonwobble substitutions cannot necessarily be generalized across different switches.

We also tested triggers with single-base insertions and deletions to identify whether the impacts of these changes were also position-dependent. Eight new TriggerA variants were synthesized, four with a C insertion and four with single nucleotide deletions at different positions in the trigger. Again, activation of SwitchA tended to be more robust to insertions and deletions in the toehold-binding region of the trigger compared with the stem-binding region (Figure 1E). In general, SwitchA also shows more robustness to deletions in triggers than to insertions at the same positions. For TriggerB and SwitchB, robustness of activation to deletions in the toehold region and sensitivity to deletions in the stem region were similar to that of SwitchA, though trends in the impacts of insertions were less clear. (Figure S5).

**Figure 2:**
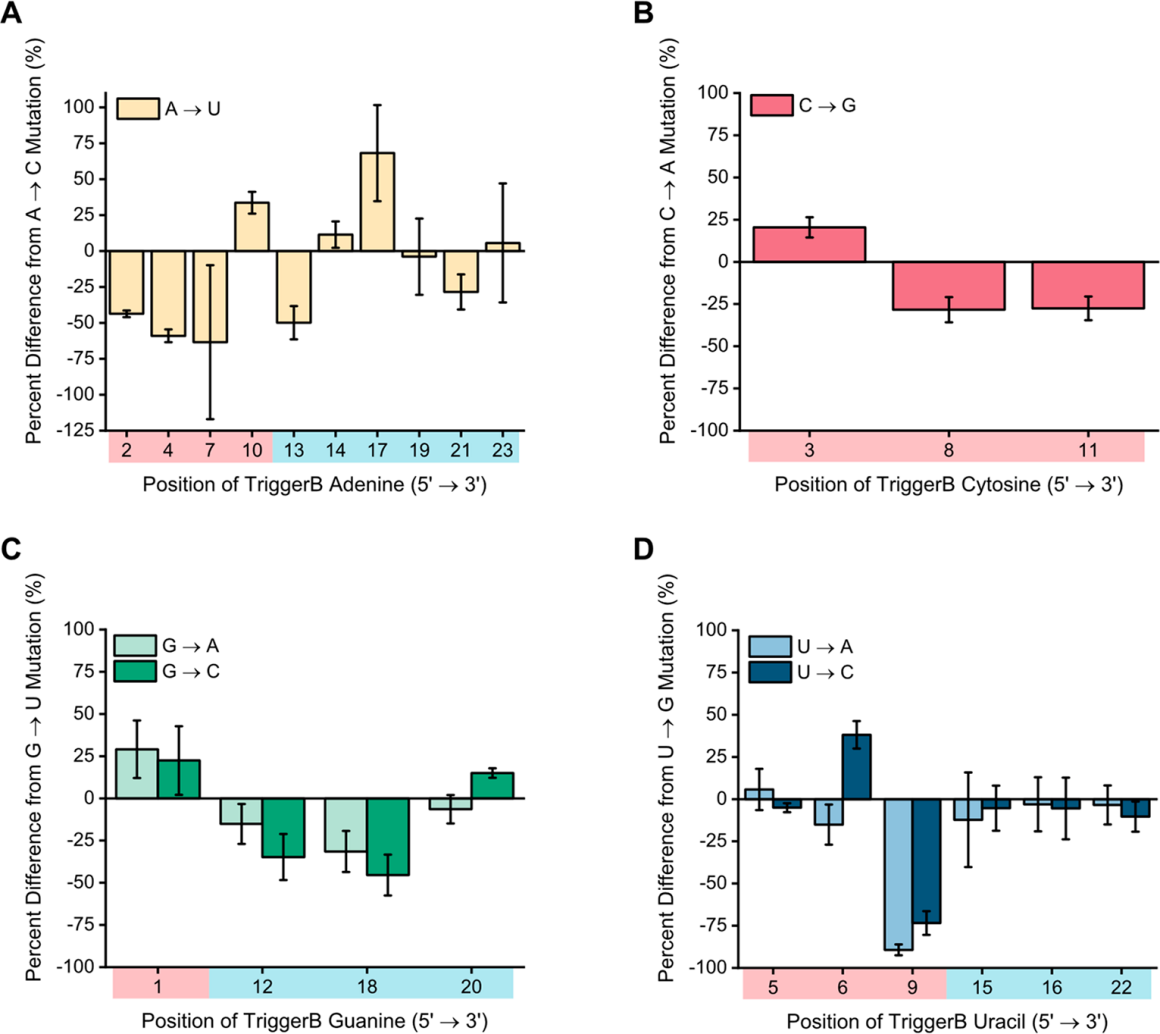
Different nucleotide substitution types can have different impacts even at the same position in the trigger. Plotted are the percent differences in GFP expressed from SwitchB when using each possible nonwobble substitution at a given position in TriggerB compared with an arbitrarily selected baseline nonwobble substitution (*y* axis) at the same position. For (A) A, (B) C, (C) G, and (D) U mutations, positions in the stem region almost always have different activation for different mutations, while the toehold regions are more likely to exhibit little difference in activation between mutations. Shading of the *x* axes indicates positions within either the stem-binding region (red) or toehold-binding region (blue). Error bars represent the standard deviation of the percent difference in activation between any pair of mutations at the same position, calculated from propagating the error from raw GFP measurements.

We then sought to characterize the remaining types of nonwobble substitutions not tested in Figure 1. (The TriggerA consensus sequence does not contain any uracil bases, so not every substitution could be tested for this switch and trigger pair.) Figure 2 shows the percent difference in SwitchB output when using triggers with one of the two or three possible types of nonwobble mutations made at the same position, all relative to a single one of those types used as a baseline (*y* axis). At most positions, there is a significant difference in SwitchB activation between the types of mutations (as indicated by the position of error bars relative to the *x* axis or relative to each other at the same position). However, there are a few positions at which the different types of mutations did not show significantly different activation levels (that is, 0% difference), and these mostly occurred in the toehold-binding region. Of all possible stem-binding region substitutions, 84% (16 out of 19) led to significantly different levels of activation for SwitchB, whereas only 42% (10 out of 24) of all possible toehold-binding region substitutions led to significant differences in SwitchB activation (Figure S6). The same trend was also observed for SwitchA (Figures S7 and S8); 88% of the stem-binding region substitutions led to significant differences in SwitchA activation while 71% of the toehold-binding region substitutions led to significant differences in SwitchA activation.

**Figure 3:**
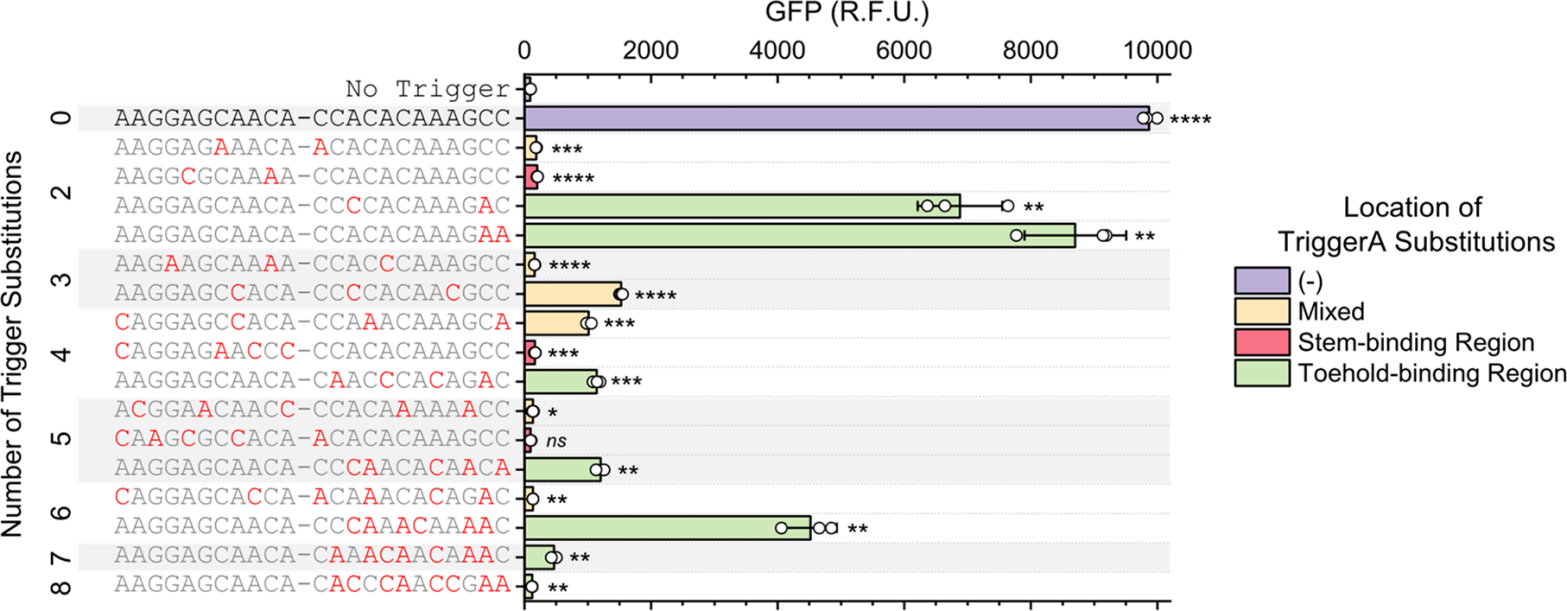
Switches can be robust to multiple mutations in the toehold-binding region of truncated triggers. GFP expression was greater when the same number of mutations was located only in the toehold-binding region (green bars) compared with triggers with mutations located in both regions (yellow bars) or exclusively in the stem-binding region (red bars). TriggerA sequence is shown on the left, where the hyphen indicates the divide between the stem and toehold regions and the red nucleotides represent mutations in each trigger variant. Error bars represent the standard deviation of technical triplicates (white circles). Asterisks indicate a significant difference between a sample and the no trigger condition, as determined by the results of a two-tailed *t* test (****P < 0.0001, ***P < 0.001, **P < 0.01, *P < 0.05).

While these single-mutation results were useful, to assess crosstalk it was also important to test the impacts of multiple mutations in a single trigger on switch activation. Sixteen new TriggerA variants that contained two to eight mutations were synthesized and tested for their ability to activate SwitchA. Not surprisingly, triggers with more mutations generally tend to induce a weaker response from the switch compared with the consensus activation (Figure 3). However, SwitchA was more robust to triggers with multiple toehold-binding region mutations compared with triggers with multiple stem-binding region mutations. For example, a trigger with four mutations in the toehold-binding region resulted in 10.7% of consensus activation, while a trigger with four mutations in the stem-binding region resulted in only 0.8% of consensus activation. Similarly, a trigger with five toehold-binding region mutations resulted in 11.4% of consensus activation, while the tested trigger with five stem-binding region mutations did not induce significant activation from the switch.

The TriggerA variant with six toehold-binding region mutations showed a surprisingly strong response of 45% of consensus activation, thereby warranting additional investigation. We tested six new TriggerA variants that each contained a subset of five of those six mutations to potentially identify a specific position responsible for the strong activation. The TriggerA variant with six mutations induced a higher response than all five-mutation variants (Figure S9), thereby further demonstrating how unpredictable the results of combining multiple mutations in a single trigger can be. SwitchA was not the only switch studied that yielded robustness to multiple mutations: a TriggerB variant with four mutations in the toehold-binding region resulted in GFP expression that was still 28% of consensus activation, and a TriggerD variant with four mutations in the toehold-binding region resulted in 45% of consensus activation (Figure S10). This TriggerD variant also yielded greater expression than a different TriggerD variant with only three toehold-binding region mutations.

**Figure 4:**
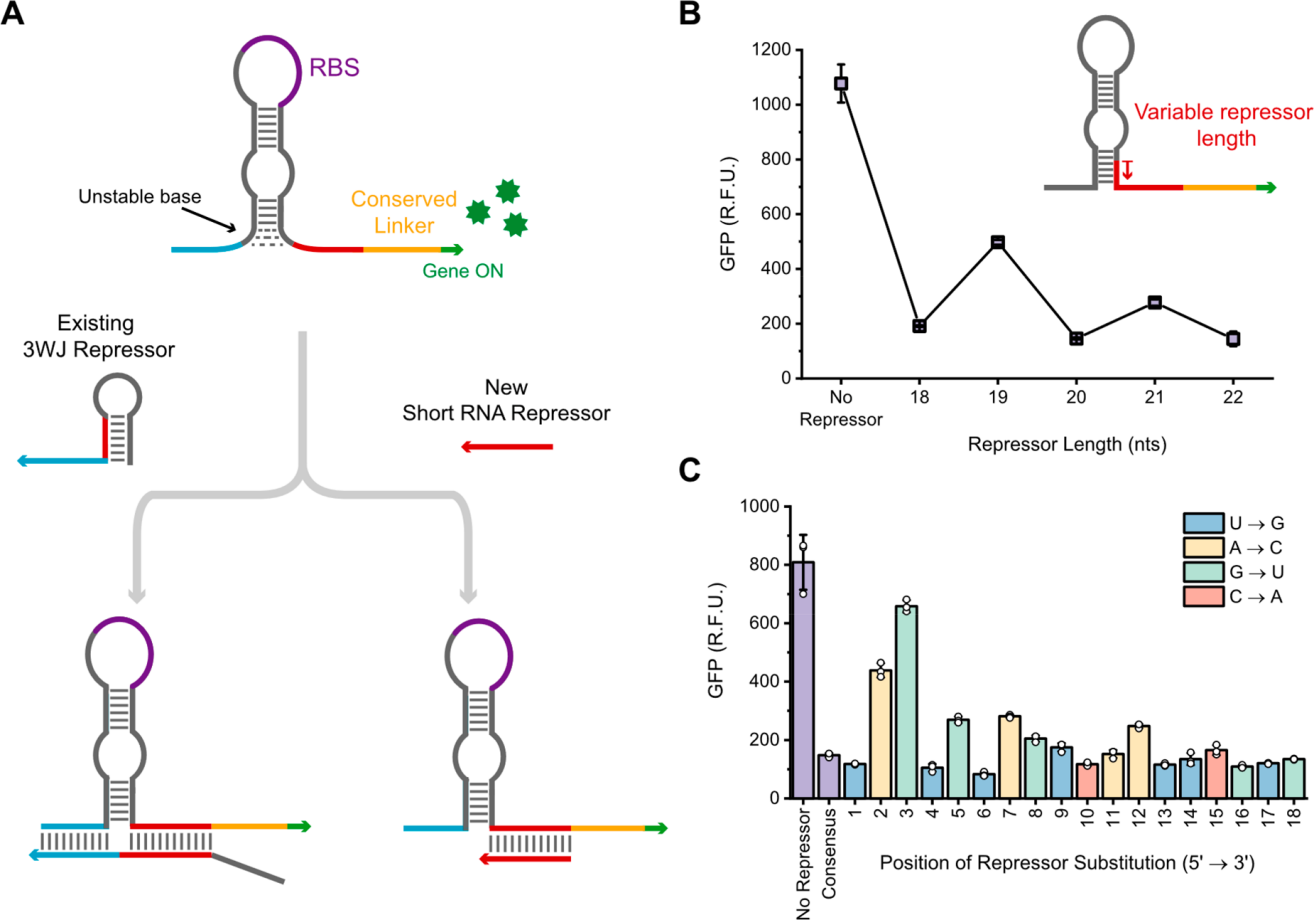
Translational repression can be implemented via the binding of short RNA molecules between the RBS and conserved linker sequence of switch mRNA. (A) Schematic showing mechanism of repression of the existing 3WJ repressor and our proposed RNA repressor. (B) Impact of repressor length on repression efficiency. (C) The impacts of single point substitutions at every position of the 18-nt repressor. Error bars represent the standard deviation of technical triplicates (white circles). .

Beyond these short, truncated trigger activators, we also sought to establish the potential for short RNA sequences to serve as repressors. The ability to induce both activation and repression from the same class of short target molecules could be valuable for the design and implementation of complex genetic circuits. To date, three approaches have been reported that use toehold-mediated interactions with trigger-length sequences as translational repressors.^22,23^ One of these designs, the three-way junction (3WJ) repressor, has already been tested with relatively short repressor sequences; the optimized 3WJ design uses a 45-nt RNA repressor but has also been characterized with repressors as short as 40 nt containing an 18-nt interaction region. Because of the simple design of the 3WJ format and its demonstrated success with short RNA repressors, we sought to use this 3WJ design as the basis for repression with even shorter sequences. To decrease the repressor length to less than 23 nt, we hypothesized that, rather than requiring a three-way junction, an RNA oligo that binds entirely after the stem region and RBS could inhibit translation by creating a stable double-stranded RNA that would dislodge the ribosome and inhibit translation (Figure 4A). Such an approach could have some advantages over previously reported formats because it relies less on the stable formation of complex RNA secondary structures between switch and repressor.

To test this approach, we designed five RNA repressors ranging from 18 to 22 nt to bind downstream of the RBS and immediately before the conserved linker sequence. The shortest repressor length we tested was 18 nt since that was the distance between the base of the stem and the conserved linker sequence. The 18-nt repressor is the only repressor that does not pair with nucleotides that fold into the base of the stem. We found that RNA repressors as short as 18 nt induced a 5.6-fold reduction in GFP expression (Figure 4B). The 20- and 22-nt repressors yielded similar levels of repression. The 19- and 21-nt repressors were less effective but still caused significant reductions in GFP expression. Further investigation needs to be done to verify if the cause of the inconsistencies in repression levels from repressors of varying length is sequence- or structure-dependent.

Next, using the 18-nt repressor, we used single point substitutions at every position to test the robustness of this repressor construct (Figure 4C). The impacts of point substitutions throughout the length of the repressor do not have an obvious position dependence—in contrast with the strong regional dependence of toehold switch activators—although there might be more robustness to mutations in the 3′ end of the repressor (the current set of data is not sufficiently generalizable to draw any concrete conclusions). If true, this could be due to the lack of complex RNA secondary structure between the base of the stem and the start codon, which would be consistent with the robustness to mutations in the simple secondary structure region for toehold binding in the toehold switch activator system.

## Conclusions

The results reported here support the feasibility of using reliable, widely used toehold switch design criteria for CFE sensors of short nucleotide sequences. Enabling of the use of RNA toehold switches with triggers much shorter than those previously reported could have significant impact on the development of diagnostic biosensors, potentially including the measurement of miRNA-length sequences. Mutations in these short trigger sequences have a strongly position-dependent impact on switch activation. Switch activation is generally robust to mutations in the toehold-binding region of the trigger, with as many as seven mutations in the toehold-binding region still allowing significant activation. Sensitivity to mutations in the stem region was consistently observed, which suggests that this region would be the source of specificity for the activation of switches by short triggers. We also reported a new approach for using 18- to 22-nt RNA sequences as translational repressors. By avoiding complex secondary structure formation and branch migration during trigger binding, these RNA repressors could have less stringent design requirements compared with existing RNA toehold repressor designs. These repressors also may be more robust to single base pair mismatches compared with toehold switch activators.

To the best of our knowledge, this is the first report to characterize the impacts of site-specific trigger mutations on cognate switch activation with the widely used Series B toehold switch design. A recent report presented a new RNA toehold switch design, named SNIPR, that excludes the stem bubble of the Series B design and uses nonconsecutive binding of an 82-nt RNA trigger for differentiation of single point mutations in RNA sequence.^24^ We have shown here that the Series B toehold switch can also be designed to differentiate single point mutations and even do so with 22 to 23-nt triggers. We also for the first time report the impacts of multiple specific mutations on a single trigger on switch activation. We demonstrate with three different switch-trigger pairs that the combinatorial effect of multiple mutations is difficult to predict and likely dependent on the sequence of the switch itself. Nonetheless, we found that switches remained robust to triggers with many mutations in the toehold-binding region—a surprising finding given that standard toehold switches were designed to be, and are often considered to be, fairly specific for their cognate triggers.

While our work is a significant step toward the development of toehold switch-based miRNA biosensors, the impacts of our findings go beyond that application. miRNA sensing at clinically relevant levels remains an outstanding challenge; the limits of detection of the approaches investigated here were not considered in this effort to demonstrate proof of principle and to characterize robustness. However, beyond miRNA, our identification of highly mutation-sensitive trigger regions could improve toehold switch sensor design to reduce crosstalk with known homologous off-target RNAs of any length, such as distinguishing between different viral strain variants. At the same time, the identification of robustness to mutations in certain regions could help enable the development of toehold switches that are intentionally permissive of mismatches, perhaps for applications like the profiling of hypervariable regions of 16S rRNA^25^ where a switch could report out on the presence of multiple species in a given family of organisms. Taken together, this mapping of the position-dependent impacts of truncated trigger point mutations on toehold switch activation could lead to the improvement of existing sensors, as well as the development of innovative new sensors, though further investigation is warranted to support the generalizability of trends identified here.

## Methods

### Bacterial Strains and Plasmid Preparation

DNA oligonucleotides for cloning and sequencing were synthesized by Eurofins Genomics. Plasmids expressing toehold switches were cloned using blunt-end ligation into plasmid backbone pJL1. *Escherichia coli* strain DH10β was used for all cloning and plasmid preparations. Isolated colonies were grown overnight in LB medium with kanamycin sulfate (33 μg/mL). Plasmid DNA from overnight cultures was purified using EZNA mini prep columns (OMEGA Bio-Tek). Plasmid sequences were verified with Sanger DNA sequencing (Eurofins Genomics). Sequence-confirmed plasmids were then purified using EZNA midiprep columns (OMEGA Bio-Tek), followed by isopropanol and ethanol precipitation. The purified DNA pellet was reconstituted in elution buffer, measured on a Nanodrop 2000 for concentration, and stored at −20 °C until use. *E. coli* strain BL21 Star (DE3) ΔlacIZYA was created by lambda red recombination^26^ and used for in-house cell-free lysate preparation.

### Toehold Switch and Trigger Variant Preparation

Toehold switches were designed using NUPACK with the Series B toehold switch design^8^ and cloned into a pJL1 plasmid containing a superfolder green fluorescent protein (sfGFP) reporter. Trigger consensus sequences were also cloned into a pJL1 plasmid excluding the T7 promoter and the sfGFP gene. All triggers (except for those used in Figure S1) were expressed from linear DNA expression templates. Linear expression templates^27^ were synthesized via PCR using Q5 DNA polymerase (New England Biolabs). After PCR amplification, all products were run on a 1 w/v % agarose gel to verify successful amplification of targets and then purified using a PCR purification kit (Omega Bio-Tek).

### Cell-Free Reactions

The cell-free reaction composition was as previously described by Kwon and Jewett.^28^ Details on the crude cell-free lysate preparation are given in the Supporting Information. All reactions (except those in Figure S1) used either 1 nM of plasmid expressing the toehold switch (activators) or 0.02 nM of plasmid expressing the toehold switch (repressor) along with 25 nM of linear DNA expression template expressing the trigger. All reactions using linear DNA expression templates were supplemented with 10 μM of Chi6 DNA oligos to inhibit DNA degradation during the reaction.^29^ All reactions were assembled on ice and incubated without shaking at 37 °C for 3 h. Each cell-free reaction mixture had a volume of 10 μL and was pipetted into a clear-bottomed 384-well plate for fluorescence measurement. GFP measurement used 485 and 510 nm for excitation and emission, respectively, with the gain set to 75. Plates were sealed with a transparent adhesive film to prevent evaporation.

## Supporting Information Available

Methods for cell-free lysate preparation; Figures S1–S10 show schematic comparison of Series A and Series B toehold switches, activation of Series B toehold switches with truncated triggers, position-specific impacts of trigger substitutions across all positions using SwitchA and SwitchB, impact of TriggerB wobble substitutions on SwitchB activation, impacts of TriggerB insertions and deletions on SwitchB activation, raw GFP data used to calculate the percent differences presented in Figure 2, percent differences in GFP expressed from SwitchA using each possible nonwobble substitution at every trigger position, raw GFP data used to calculate the percent differences presented in Figure S7, unpredictable impacts of multiple mutations in the same trigger on switch activation, and the impacts of multiple mutations in the same trigger using SwitchB and SwitchD; and Tables S1–S3 describing the statistical analysis of Figure S2, all plasmid sequences used in this study, and all PCR primer sequences used in this study (PDF)

## References

[ref1] Voyvodic P. L.; Bonnet J. Cell-Free Biosensors for Biomedical Applications. Curr. Opin. Biomed. Eng. 2020, 13, 9–15. 10.1016/j.cobme.2019.08.005.

[ref2] McNerney M. P.; Piorino F.; Michel C. L.; Styczynski M. P. Active Analyte Import Improves the Dynamic Range and Sensitivity of a Vitamin B12 Biosensor. ACS Synth. Biol. 2020, 9 (2), 402–411. 10.1021/acssynbio.9b00429. 31977200 PMC7186122

[ref3] Voyvodic P. L.; Pandi A.; Koch M.; Conejero I.; Valjent E.; Courtet P.; Renard E.; Faulon J.-L.; Bonnet J. Plug-and-Play Metabolic Transducers Expand the Chemical Detection Space of Cell-Free Biosensors. Nat. Commun. 2019, 10 (1), 1697 10.1038/s41467-019-09722-9. 30979906 PMC6461607

[ref4] McNerney M. P.; Zhang Y.; Steppe P.; Silverman A. D.; Jewett M. C.; Styczynski M. P. Point-of-Care Biomarker Quantification Enabled by Sample-Specific Calibration. Sci. Adv. 2019, 5 (9), eaax4473 10.1126/sciadv.aax4473. 31579825 PMC6760921

[ref5] Zhang Y.; Kojima T.; Kim G.-A.; McNerney M. P.; Takayama S.; Styczynski M. P. Protocell Arrays for Simultaneous Detection of Diverse Analytes. Nat. Commun. 2021, 12 (1), 5724 10.1038/s41467-021-25989-3. 34588445 PMC8481512

[ref6] Pardee K.; Green A. A.; Ferrante T.; Cameron D. E.; DaleyKeyser A.; Yin P.; Collins J. J. Paper-Based Synthetic Gene Networks. Cell 2014, 159 (4), 940–954. 10.1016/j.cell.2014.10.004. 25417167 PMC4243060

[ref7] Green A. A.; Silver P. A.; Collins J. J.; Yin P. Toehold Switches: De-Novo-Designed Regulators of Gene Expression. Cell 2014, 159 (4), 925–939. 10.1016/j.cell.2014.10.002. 25417166 PMC4265554

[ref8] Pardee K.; Green A. A.; Takahashi M. K.; Braff D.; Lambert G.; Lee J. W.; Ferrante T.; Ma D.; Donghia N.; Fan M.; Daringer N. M.; Bosch I.; Dudley D. M.; O’Connor D. H.; Gehrke L.; Collins J. J. Rapid, Low-Cost Detection of Zika Virus Using Programmable Biomolecular Components. Cell 2016, 165 (5), 1255–1266. 10.1016/j.cell.2016.04.059. 27160350

[ref9] Hunt J. P.; Zhao E. L.; Free T. J.; Soltani M.; Warr C. A.; Benedict A. B.; Takahashi M. K.; Griffitts J. S.; Pitt W. G.; Bundy B. C. Towards Detection of SARS-CoV-2 RNA in Human Saliva: A Paper-Based Cell-Free Toehold Switch Biosensor with a Visual Bioluminescent Output. New Biotechnol 2022, 66, 53–60. 10.1016/j.nbt.2021.09.002. PMC8452453 34555549

[ref10] Carr A. R.; Dopp J. L.; Wu K.; Sadat Mousavi P.; Jo Y. R.; McNeley C. E.; Lynch Z. T.; Pardee K.; Green A. A.; Reuel N. F. Toward Mail-in-Sensors for SARS-CoV-2 Detection: Interfacing Gel Switch Resonators with Cell-Free Toehold Switches. ACS Sens 2022, 7 (3), 806–815. 10.1021/acssensors.1c02450. 35254055

[ref11] Cissell K. A.; Shrestha S.; Deo S. K. MicroRNA Detection: Challenges for the Analytical Chemist. Anal. Chem. 2007, 79 (13), 4754–4761. 10.1021/ac0719305.

[ref12] Jovanovic M.; Hengartner M. O. MiRNAs and Apoptosis: RNAs to Die For. Oncogene 2006, 25 (46), 6176–6187. 10.1038/sj.onc.1209912. 17028597

[ref13] Cortez M. A.; Bueso-Ramos C.; Ferdin J.; Lopez-Berestein G.; Sood A. K.; Calin G. A. MicroRNAs in Body Fluids—the Mix of Hormones and Biomarkers. Nat. Rev. Clin. Oncol. 2011, 8 (8), 467–477. 10.1038/nrclinonc.2011.76. 21647195 PMC3423224

[ref14] Halushka M. K.; Fromm B.; Peterson K. J.; McCall M. N. Big Strides in Cellular MicroRNA Expression. Trends Genet. TIG 2018, 34 (3), 165–167. 10.1016/j.tig.2017.12.015. 29361313 PMC5834366

[ref15] Quan X.; Ji Y.; Zhang C.; Guo X.; Zhang Y.; Jia S.; Ma W.; Fan Y.; Wang C. Circulating MiR-146a May Be a Potential Biomarker of Coronary Heart Disease in Patients with Subclinical Hypothyroidism. Cell. Physiol. Biochem. 2018, 45 (1), 226–236. 10.1159/000486769. 29357324

[ref16] Ben-Dov I. Z.; Tan Y.-C.; Morozov P.; Wilson P. D.; Rennert H.; Blumenfeld J. D.; Tuschl T. Urine MicroRNA as Potential Biomarkers of Autosomal Dominant Polycystic Kidney Disease Progression: Description of MiRNA Profiles at Baseline. PLoS One 2014, 9 (1), e86856 10.1371/journal.pone.0086856. 24489795 PMC3906110

[ref17] Ali Ahmed E.; Abd El-Basit S. A.; Mohamed M. A.; Swellam M. Clinical Role of MiRNA 29a and MiRNA 335 on Breast Cancer Management: Their Relevance to MMP2 Protein Level. Arch. Physiol. Biochem. 2022, 128, 1058–1065. 10.1080/13813455.2020.1749085. 32267166

[ref18] Calin G. A.; Dumitru C. D.; Shimizu M.; Bichi R.; Zupo S.; Noch E.; Aldler H.; Rattan S.; Keating M.; Rai K.; Rassenti L.; Kipps T.; Negrini M.; Bullrich F.; Croce C. M. Frequent Deletions and Down-Regulation of Micro- RNA Genes MiR15 and MiR16 at 13q14 in Chronic Lymphocytic Leukemia. Proc. Natl. Acad. Sci. U. S. A. 2002, 99 (24), 15524–15529. 10.1073/pnas.242606799. 12434020 PMC137750

[ref19] Lu J.; Getz G.; Miska E. A.; Alvarez-Saavedra E.; Lamb J.; Peck D.; Sweet-Cordero A.; Ebert B. L.; Mak R. H.; Ferrando A. A.; Downing J. R.; Jacks T.; Horvitz H. R.; Golub T. R. MicroRNA Expression Profiles Classify Human Cancers. Nature 2005, 435 (7043), 834–838. 10.1038/nature03702. 15944708

[ref20] Tribolet L.; Kerr E.; Cowled C.; Bean A. G. D.; Stewart C. R.; Dearnley M.; Farr R. J. MicroRNA Biomarkers for Infectious Diseases: From Basic Research to Biosensing. Front. Microbiol. 2020, 11, 1197 10.3389/fmicb.2020.01197. 32582115 PMC7286131

[ref21] Wang S.; Emery N. J.; Liu A. P. A Novel Synthetic Toehold Switch for MicroRNA Detection in Mammalian Cells. ACS Synth. Biol. 2019, 8 (5), 1079–1088. 10.1021/acssynbio.8b00530. 31039307

[ref22] Kim J.; Zhou Y.; Carlson P. D.; Teichmann M.; Chaudhary S.; Simmel F. C.; Silver P. A.; Collins J. J.; Lucks J. B.; Yin P.; Green A. A. De Novo-Designed Translation-Repressing Riboregulators for Multi-Input Cellular Logic. Nat. Chem. Biol. 2019, 15 (12), 1173–1182. 10.1038/s41589-019-0388-1. 31686032 PMC6864284

[ref23] Carlson P. D.; Glasscock C. J.; Lucks J. B. De Novo Design of Translational RNA Repressors. bioRxiv, December 19, 2018, 501767. 10.1101/501767.

[ref24] Hong F.; Ma D.; Wu K.; Mina L. A.; Luiten R. C.; Liu Y.; Yan H.; Green A. A. Precise and Programmable Detection of Mutations Using Ultraspecific Riboregulators. Cell 2020, 180 (5), 1018–1032.e16. 10.1016/j.cell.2020.02.011. 32109416 PMC7063572

[ref25] Takahashi M. K.; Tan X.; Dy A. J.; Braff D.; Akana R. T.; Furuta Y.; Donghia N.; Ananthakrishnan A.; Collins J. J. A Low-Cost Paper-Based Synthetic Biology Platform for Analyzing Gut Microbiota and Host Biomarkers. Nat. Commun. 2018, 9 (1), 3347 10.1038/s41467-018-05864-4. 30131493 PMC6104080

[ref26] Datsenko K. A.; Wanner B. L. One-Step Inactivation of Chromosomal Genes in Escherichia Coli K-12 Using PCR Products. Proc. Natl. Acad. Sci. U. S. A. 2000, 97 (12), 6640–6645. 10.1073/pnas.120163297. 10829079 PMC18686

[ref27] McSweeney M. A.; Styczynski M. P. Effective Use of Linear DNA in Cell-Free Expression Systems. Front. Bioeng. Biotechnol. 2021, 9, 715328 10.3389/fbioe.2021.715328. 34354989 PMC8329657

[ref28] Kwon Y.-C.; Jewett M. C. High-Throughput Preparation Methods of Crude Extract for Robust Cell-Free Protein Synthesis. Sci. Rep. 2015, 5 (1), 8663 10.1038/srep08663. 25727242 PMC4345344

[ref29] Marshall R.; Maxwell C. S.; Collins S. P.; Beisel C. L.; Noireaux V. Short DNA Containing χ Sites Enhances DNA Stability and Gene Expression in E. Coli Cell-Free Transcription–Translation Systems. Biotechnol. Bioeng. 2017, 114 (9), 2137–2141. 10.1002/bit.26333. 28475211 PMC5522353

