# Supplementary material for: Short Activators and Repressors of RNA Toehold Switches
Source: ACS Synth Biol. 2023 Feb 21;12(3):681–8. doi: 10.1021/acssynbio.2c00641 (PMC10028691; doi:10.1021/acssynbio.2c00641)
Supplement: Supplementary file 1 — sb2c00641_si_001.pdf [file sb2c00641_si_001.pdf]

Supporting Information for

Short activators and repressors of RNA toehold switches

Megan A. McSweeney, Yan Zhang, and Mark P. Styczynski*

School of Chemical & Biomolecular Engineering, Georgia Institute of Technology, Atlanta, GA 30332, United States

*Corresponding author (email: mark.styczynski@gatech.edu)

Table of Contents

Supplemental Methods S3

Preparation of cell-free lysate S3

Figure S1 ..... S4

Schematic of trigger-mediated toehold switch activation mechanism of (A) the Series A design S4

Figure S2 ..... S5

Truncated output triggers designed by the Series B toehold switch NUPACK code demonstrate the potential for toehold switch-mediated detection of miRNA-length triggers expressed from plasmids S5

Figure S3 ..... S6

The position-specific impacts of trigger substitutions across all positions on activation of SwitchA and SwitchB are qualitatively consistent S6

Figure S4 ..... S7

The impact of TriggerB wobble substitutions on SwitchB activation compared to non-wobble substitutions was less consistent than for TriggerA and SwitchA S7

Figure S5 ..... S8

The impacts of (A) TriggerB insertions on SwitchB activation were less clear than for TriggerA, though the impacts of (B) TriggerB deletions were consistent with observations for TriggerA S8

Figure S6 ..... S9

Different substitution types in TriggerB often lead to different levels of activation S9

Figure S7 ..... S10

Different substitution types in TriggerA often lead to different levels of activation S10

Figure S8 ..... S11

Different substitution types in TriggerA often lead to different levels of activation S11

Figure S9 ..... S12

Combining multiple mutations in the same trigger can lead to unpredictable impacts on switch activation S12

Figure S10 ..... S13

The presence of multiple trigger mutations in (A) SwitchB and (B) SwitchD can lead to non-additive effects and statistically significant activation even for high numbers of mutations in the toehold-binding region S13

Table S1 .... S14

Statistical analysis of Figure S2 .... S14

Table S2: Description of plasmids used in this study S15

Table S3: Primer sequences used in this study S19

Table S3A: Primer sequences used for TriggerA syntheses S19

Table S3B: Primer sequences used for TriggerB syntheses S22

Table S3C: Primer sequences used for TriggerC syntheses S24

Table S3D: Primer sequences used for TriggerD syntheses S24

Table S3E: Primer sequences used for Repressor syntheses S24

Table S3F: Reverse primers used for amplification S25

References: S26

Supplemental Methods

Preparation of cell-free lysate

Cellular lysate for all experiments was prepared as described by Sun et al.[1] with a few protocol modifications. Briefly, BL21 Star (DE3) ΔlacZ cells were grown in 2xYTP medium at 37 °C and 220 rpm to an optical density (OD) between 1.5-2.0, corresponding to the mid-exponential growth phase. 0.4 mM IPTG was added when the OD reached 0.4 to induce expression of T7 RNA polymerase, creating a T7 RNAP-enriched lysate. Cells were centrifuged at 2700 × g and washed via resuspension with S30A buffer (50 mM tris, 14 mM magnesium glutamate, 60 mM potassium glutamate, 2 mM dithiothreitol, and pH-corrected to 7.7 with acetic acid). These centrifugation and wash steps were repeated twice for a total of three S30A washes. After the final centrifugation, the wet cell mass was determined, and cells were resuspended in 1 mL of S30A buffer per 1 g of wet cell mass. The cellular resuspension was divided into 1 mL aliquots. Cells were lysed using a Q125 sonicator (Qsonica) at a frequency of 20 kHz and 50% of amplitude. Cells were sonicated on ice with cycles of 10 sec on and 10 sec off, delivering approximately 300–350 J, at which point the cells appeared visibly lysed. An additional 4 mM dithiothreitol was added to each tube, and the sonicated mixture was then centrifuged at 12,000 × g and 4 °C for 10 min. After centrifugation, the supernatant was removed, divided into 1 mL aliquots, and incubated at 37 °C and 220 rpm for 80 min. After this runoff reaction, the cellular lysate was centrifuged at 12,000 × g and 4 °C for 10 min. The supernatant was removed and loaded into a 10 kDa molecular weight cutoff dialysis cassette (Thermo Fisher). Lysate was dialyzed in 1 L of S30B buffer (14 mM magnesium glutamate, 60 mM potassium glutamate, 1 mM dithiothreitol, and pH-corrected to 8.2 with tris) at 4 °C for 3 h. Dialyzed lysate was removed and centrifuged at 12,000 × g and 4 °C for 10 min. The supernatant was removed, aliquoted, and stored at −80 °C for future use.

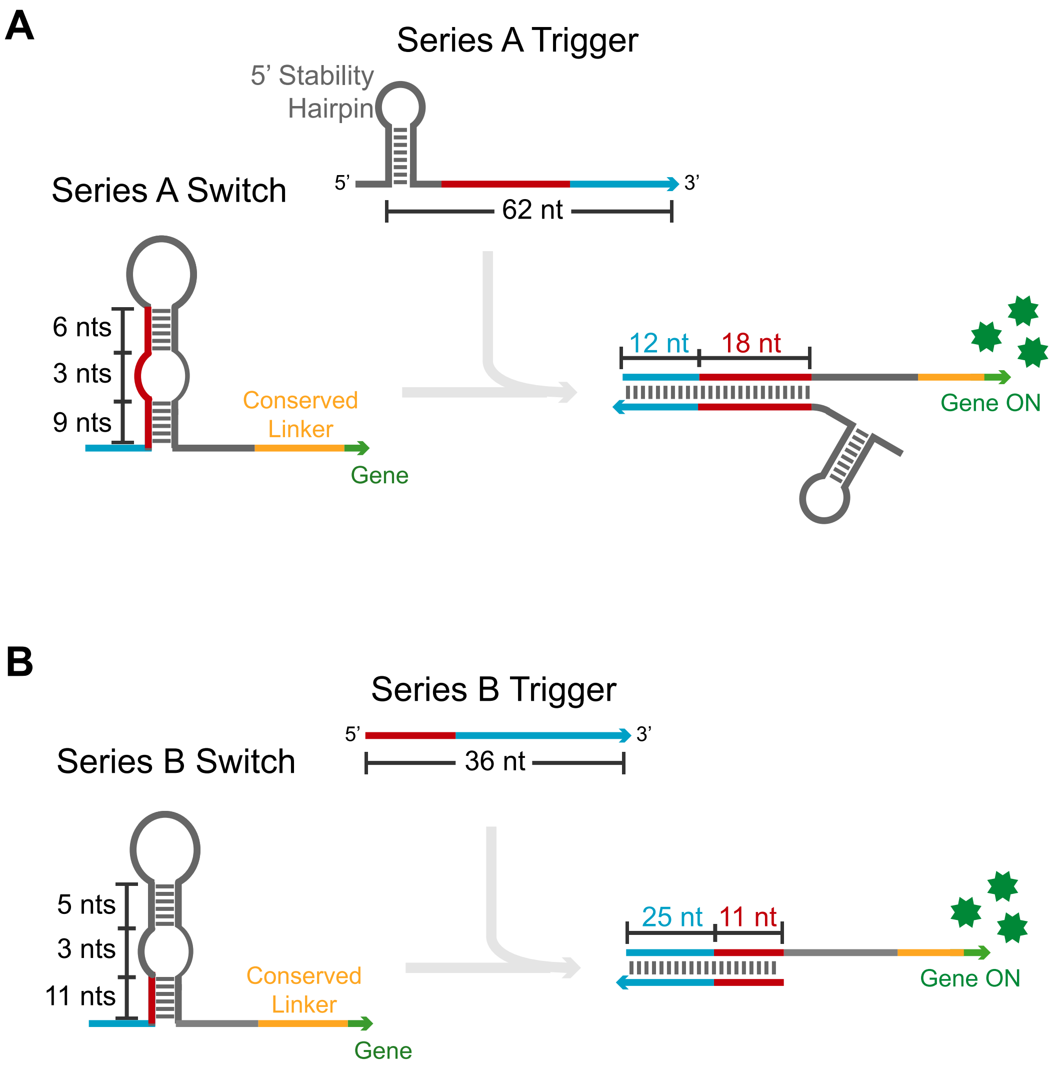

Figure S1
Schematic of trigger-mediated toehold switch activation mechanism of (A) the Series A design[2] and (B) Series B design[3]. Differences of note include the overall length of the RNA trigger, the inclusion of a 5' stability hairpin on the Series A trigger, and the length of the toehold-switch hairpin regions.

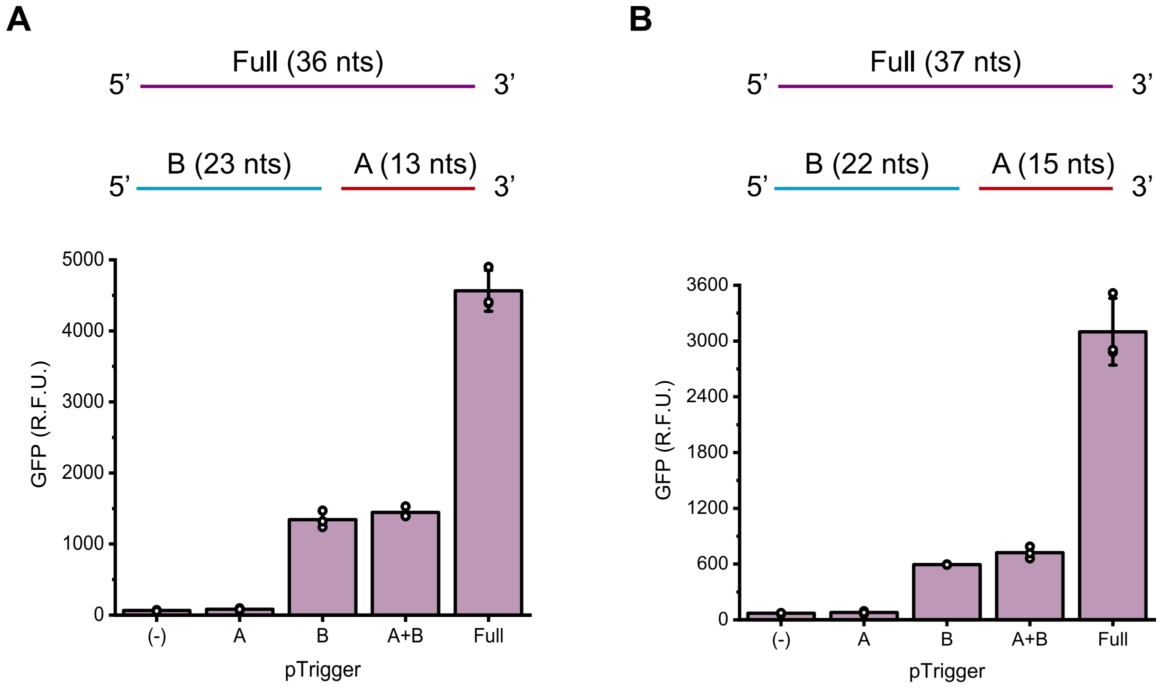

Figure S2

Truncated output triggers designed by the Series B toehold switch NUPACK code demonstrate the potential for toehold switch-mediated detection of miRNA-length triggers expressed from plasmids. Schematics above graphs indicate how the full trigger sequences were divided into shorter A and B trigger fragments for (A) SwitchA and (B) SwitchC. (-) indicates the switch-only condition in the absence of any trigger plasmid. Each reaction contains 2.5 nM of the toehold switch plasmid and 5 nM of the trigger plasmid (pTrigger). Conditions with two trigger plasmids have 5 nM of each plasmid. Error bars represent the standard deviation of technical triplicates (white circles).

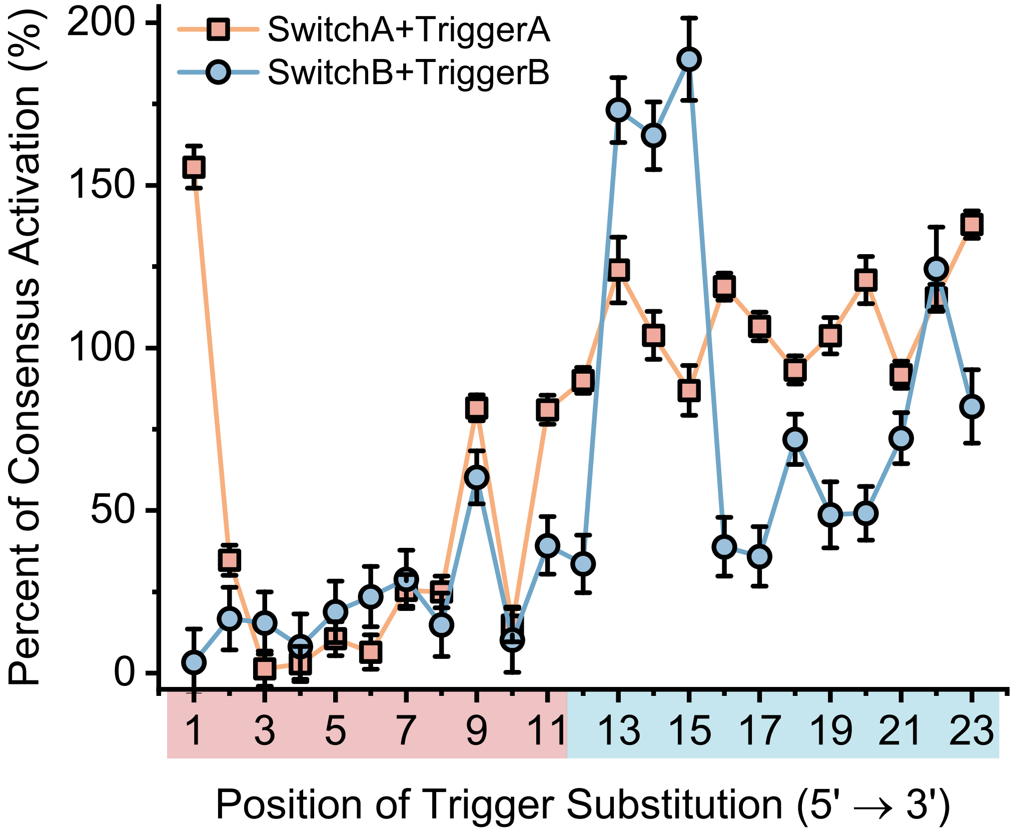

Figure S3

The position-specific impacts of trigger substitutions across all positions on activation of SwitchA and SwitchB are qualitatively consistent. Mutations in the stem-binding region have a greater impact on switch activation than mutations in the trigger-binding region, expressed as the percent of activation by the consensus truncated trigger. X axis shading indicates positions within either the stem-binding region (red) or toehold-binding region (blue). Error bars represent the standard deviation of technical triplicates.

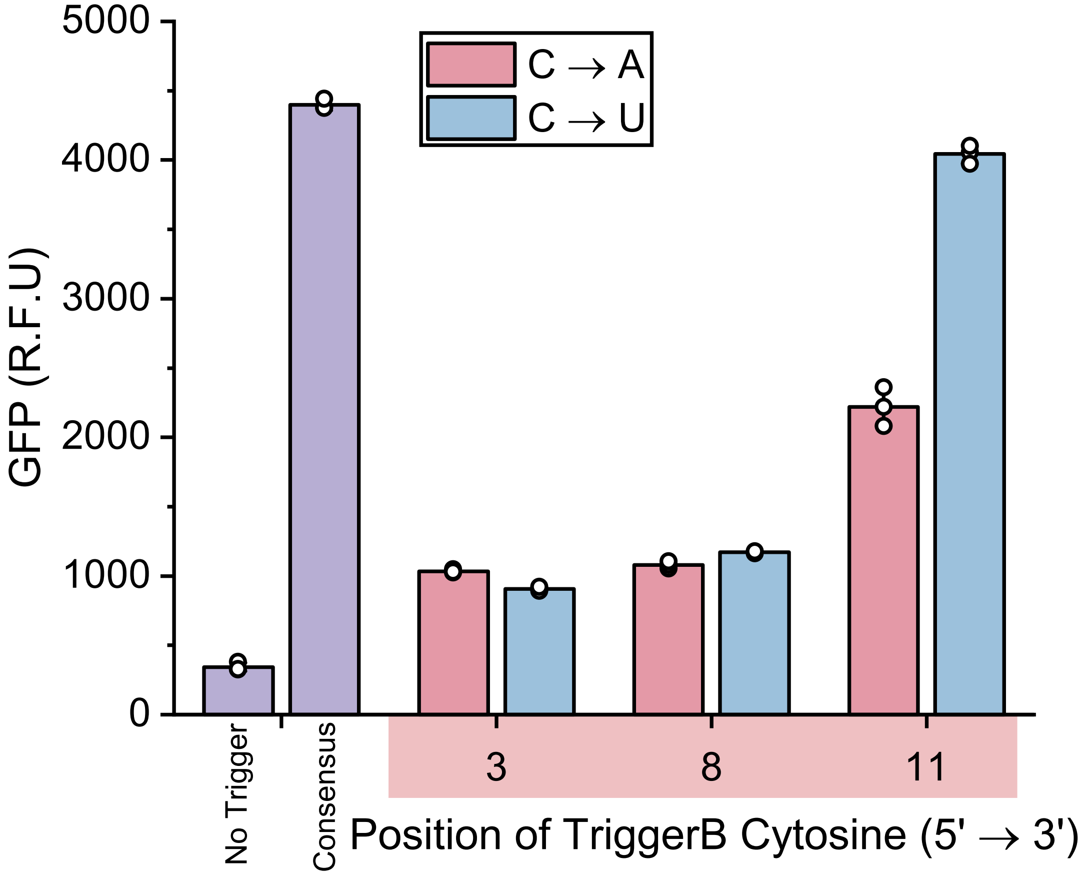

Figure S4
The impact of TriggerB wobble substitutions on SwitchB activation compared to non-wobble substitutions was less consistent than for TriggerA and SwitchA. Positions 3, 8, and 11 are all within the stem-binding region. Error bars represent the standard deviation of technical triplicates (white circles).

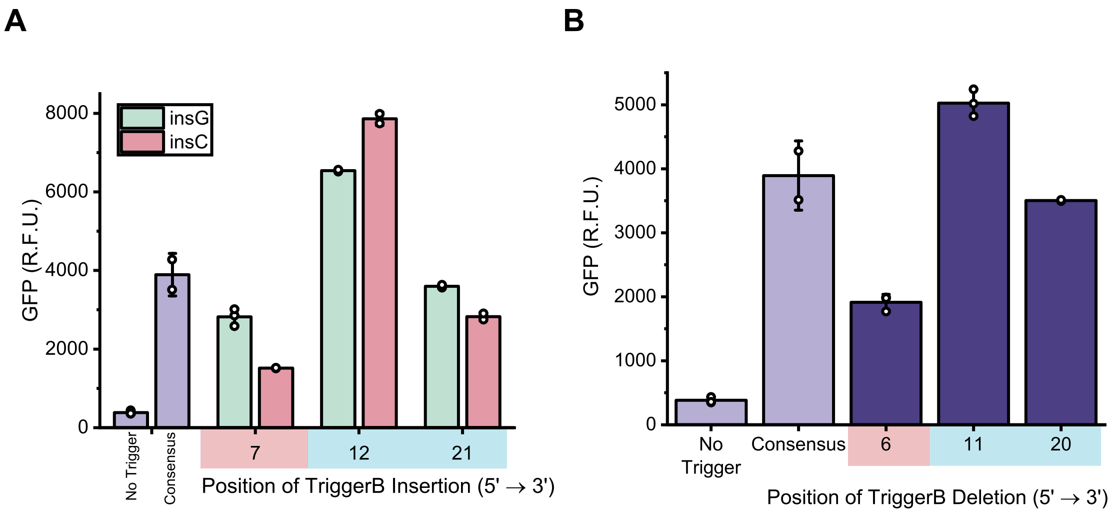

Figure S5

The impacts of (A) TriggerB insertions on SwitchB activation were less clear than for TriggerA, though the impacts of (B) TriggerB deletions were consistent with observations for TriggerA. X axis shading indicates positions within either the stem-binding region (red) or toehold-binding region (blue). Error bars represent the standard deviation of technical triplicates (white circles).

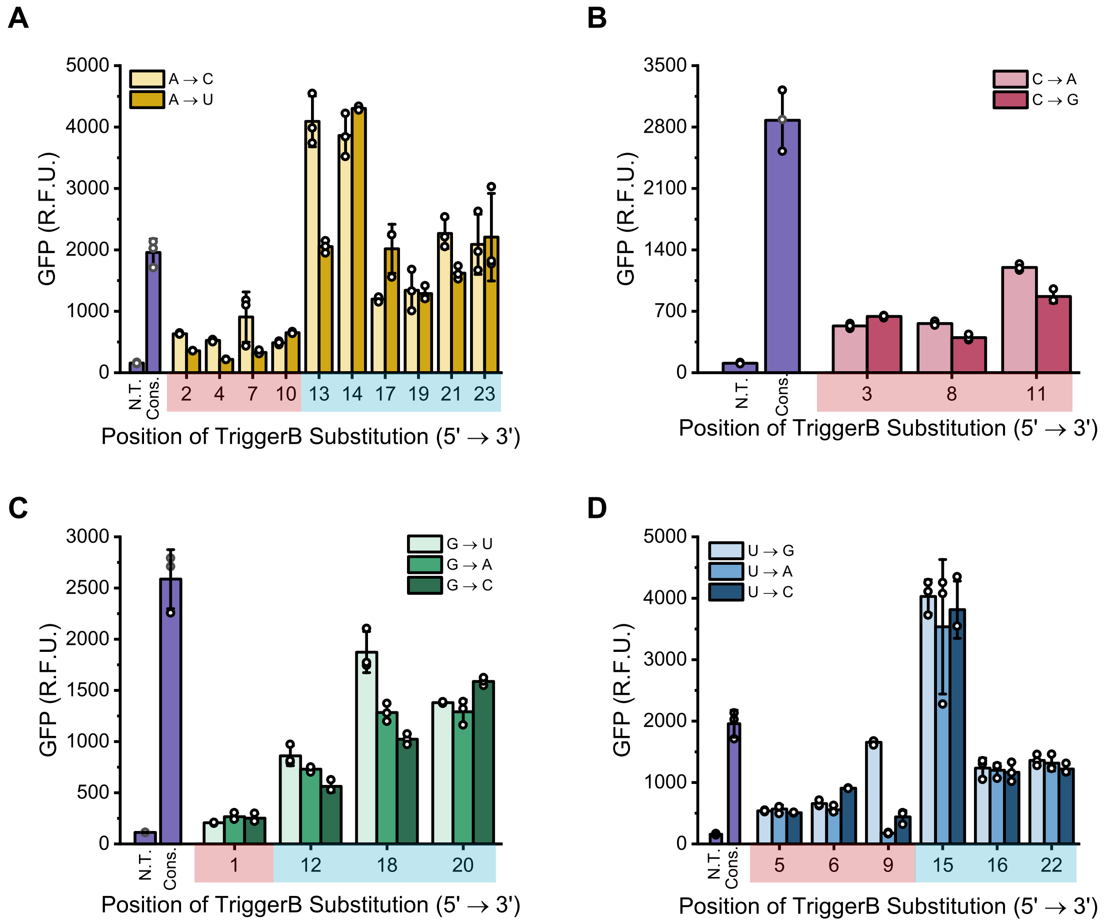

Figure S6
Different substitution types in TriggerB can lead to different levels of activation. Plotted are the GFP values used to calculate the percent differences shown in **Figure 2** for (A) A, (B) C, (C) G, and (D) U mutations. X axis shading indicates positions within either the stem-binding region (red) or toehold-binding region (blue). Error bars represent the standard deviation of technical triplicates (white circles).

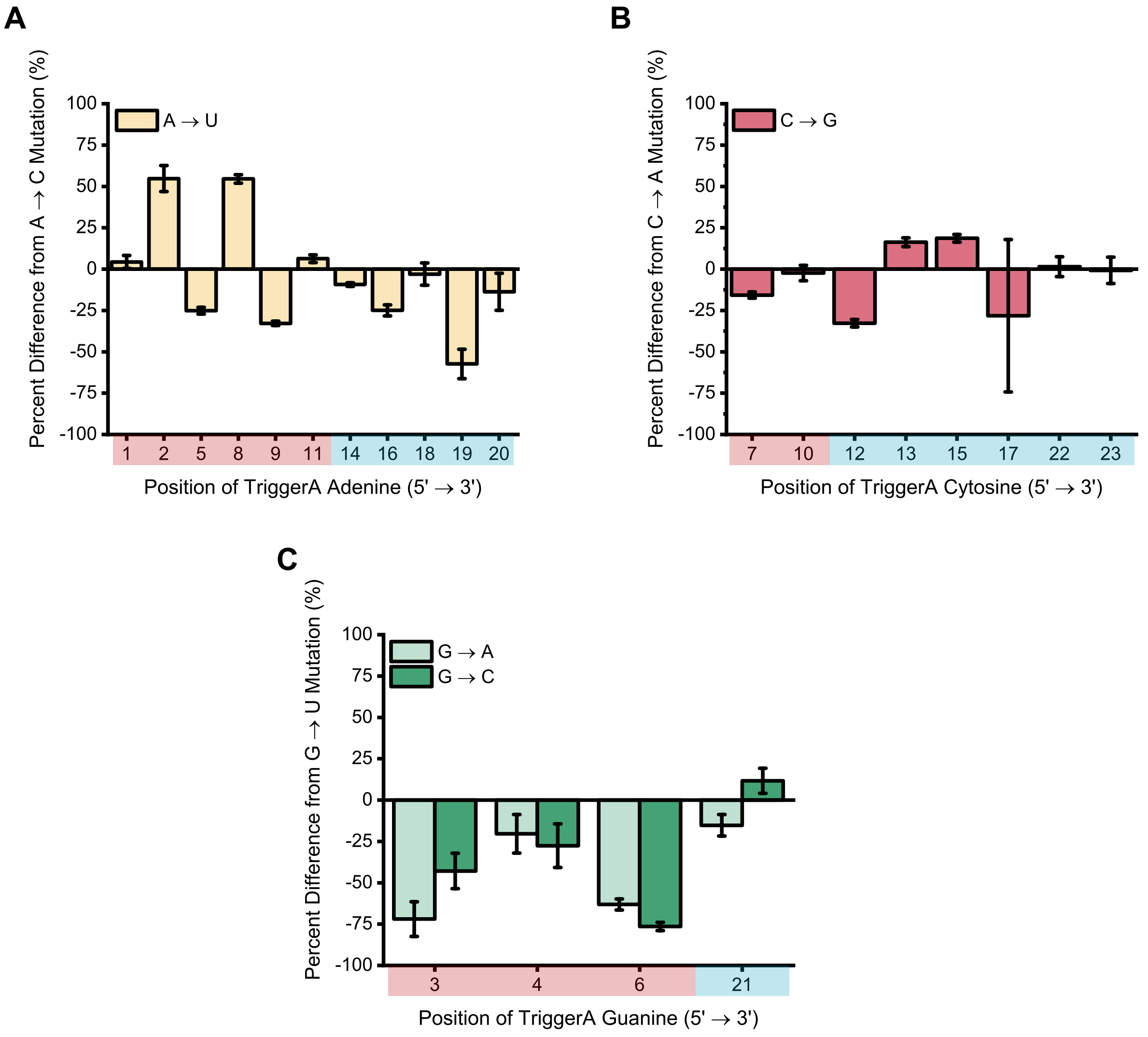

Figure S7
Different substitution types in TriggerA often lead to different levels of activation. Plotted are the percent differences in GFP expressed from SwitchA when using each possible non-wobble substitution at a given position in TriggerA compared to an arbitrarily selected baseline non-wobble substitution (y-axis) at the same position. For (A) A, (B) C, and (C) G mutations, positions in the stem region almost always have different activation for different mutations, while the toehold regions are more likely to exhibit little difference in activation between mutations. X axis shading indicates positions within either the stem-binding region (red) or toehold-binding region (blue). Error bars represent the standard deviation of the percent difference in activation between any pair of mutations at the same position.

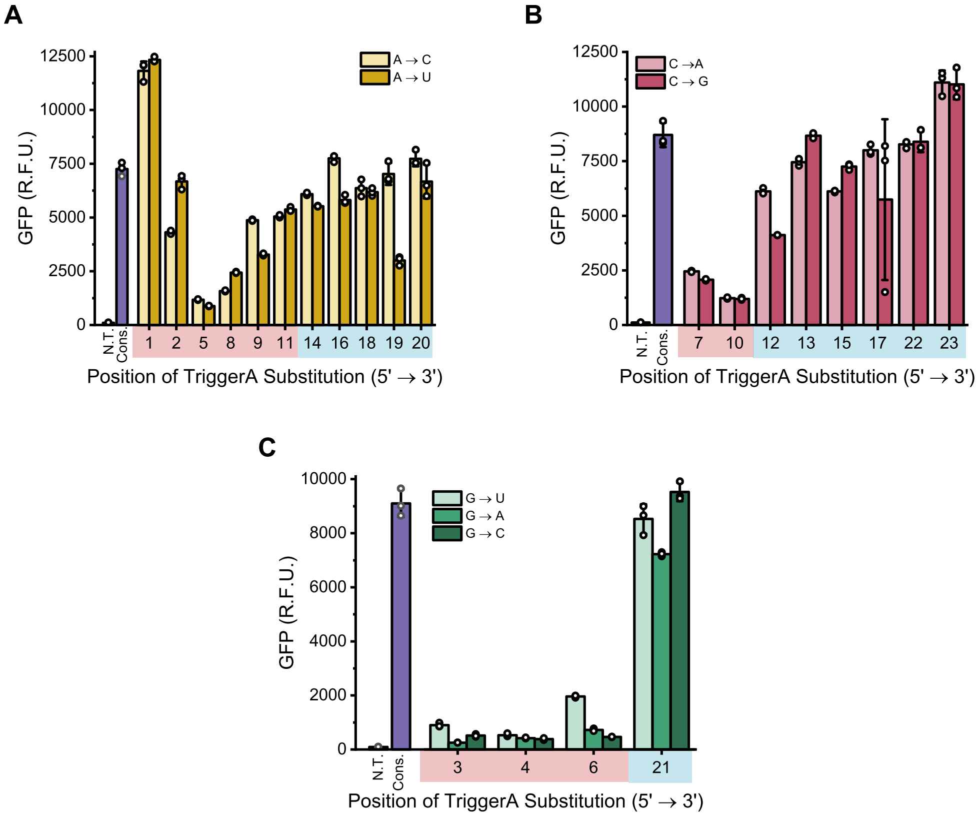

Figure S8
Different substitution types in TriggerA often lead to different levels of activation. Plotted are the GFP values used to calculate the percent differences shown in **Figure S7** for (A) A, (B) C, and (C) G mutations. X axis shading indicates positions within either the stem-binding region (red) or toehold-binding region (blue). Error bars represent the standard deviation of technical triplicates (white circles).

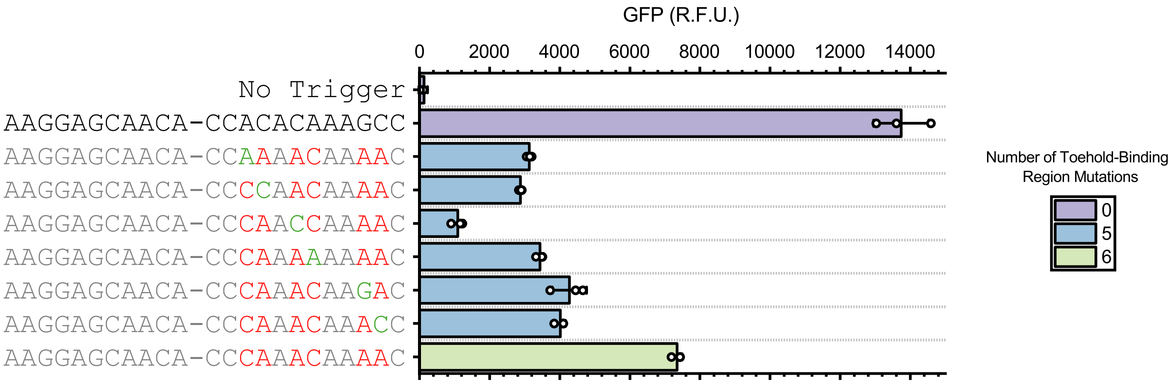

Figure S9
Combining multiple mutations in the same trigger can lead to unpredictable impacts on switch activation. TriggerA sequence is shown on the left, with the hyphen indicating the divide between the stem and toehold regions, and the red nucleotides representing mutations in each trigger variant. Green nucleotides represent consensus trigger nucleotides that are not mutated in each 5-mutation variant but are mutated in the 6-mutation variant. Error bars represent the standard deviation of technical triplicates (white circles).

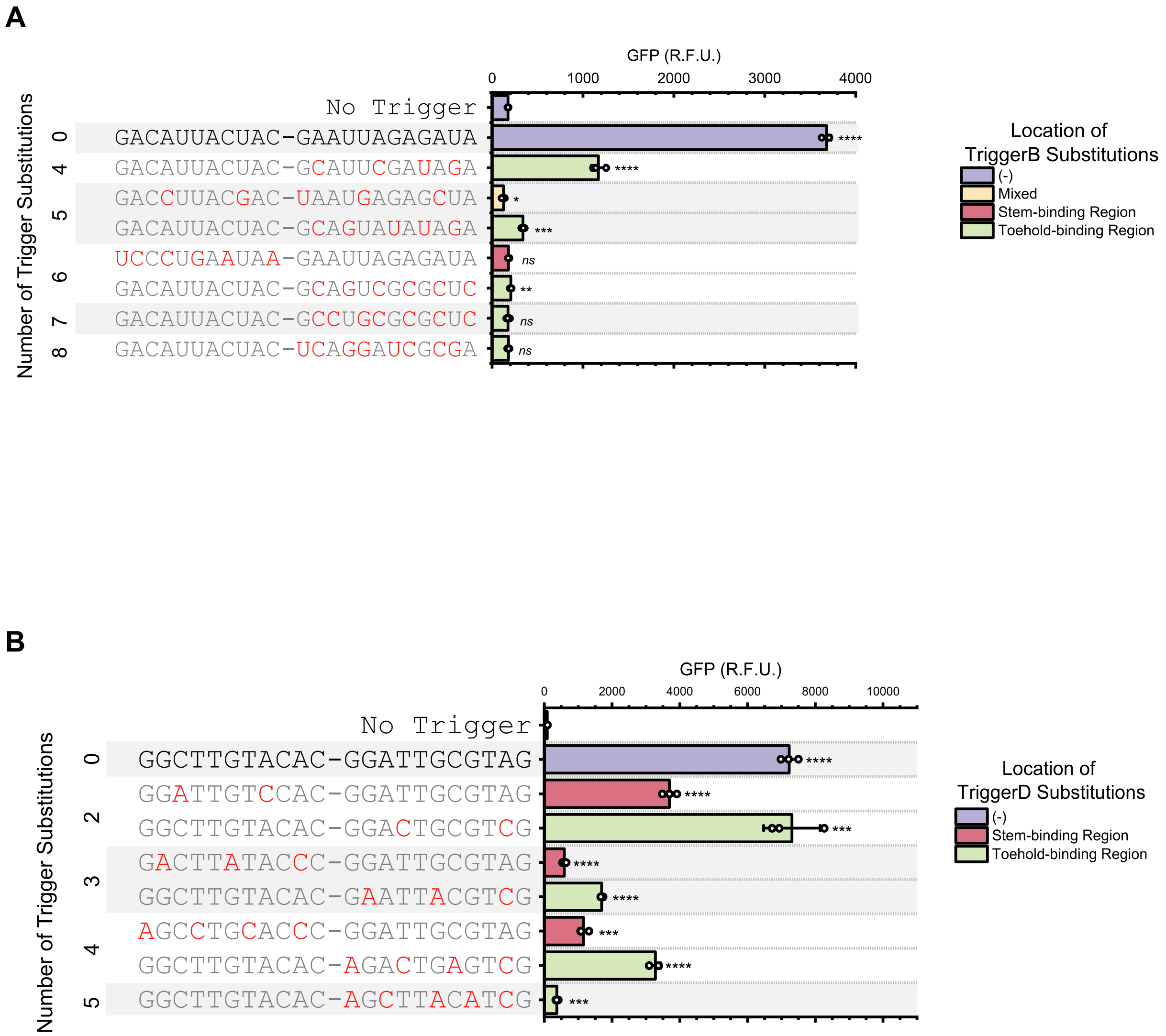

Figure S10
The presence of multiple trigger mutations in (A) SwitchB and (B) SwitchD can lead to non-additive effects and statistically significant activation even for high numbers of mutations in the toehold-binding region. Error bars represent the standard deviation of technical triplicates (white circles). Asterisks indicate a significant difference between a sample and the no trigger conditions as determined by the results of a two-tailed t-test (****$P < 0.0001$, ***$P < 0.001$, **$P < 0.01$, *$P < 0.05$).

Table S1

Statistical analysis of Figure S2

	Stem-binding region		Toehold-binding region		
	M	SD	M	SD	*p*
**SwitchA**	5548.88	6215.61	14676.8	2134.72	0.0001336
**SwitchB**	1300.70	724.277	4464.56	2583.74	0.001014

Stem-binding region = trigger positions 1-11. Toehold-binding region = trigger positions 12-23. M = arithmetic mean. SD = standard deviation. *p* is the result of a two-tailed *t*-test.

Table S2: Description of plasmids used in this study

All plasmids used in this study contain a ColE1 origin of replication and kanamycin resistance marker from pJL1.

<table>
<tr><th>pSwitchA_sfGFP</th><th>Plasmid encoding sfGFP under regulation of toehold switch A</th></tr>
<tr><td colspan="2">T7 Promoter-SwitchA-sfGFP-TrrnB-T7 Terminator

taatacgactcactataggGTTACTGTATGACCGGCTTTGTGTGGTGTTGCTCCTTGGACTTTAGAACAGAGGAGATAAAGATGAAGGAGCAACAC
AACCTGGCGGCAGCGCAAAAGatgcgtaaaggagaagaacttttcactggagttgtcccaattcttgttgaattagatggtgatgttaatgggcac
aaattttctgtccgtggagagggtgaaggtgatgctacaaacggaaaactcacccttaaatttatttgcactactggaaaactacctgttccgtgg
ccaacacttgtcactactctgacctatggtgttcaatgcttttcccgttatccggatcacatgaaacggcatgactttttcaagagtgccatgccc
gaaggttatgtacaggaacgcactatatctttcaaagatgacgggacctacaagacgcgtgctgaagtcaagtttgaaggtgatacccttgttaat
cgtatcgagttaaagggtattgattttaaagaagatggaaacattcttggacacaaactcgagtacaactttaactcacacaatgtatacatcacg
gcagacaaacaaaagaatggaatcaaagctaacttcaaaattcgccacaacgttgaagatggttccgttcaactagcagaccattatcaacaaaat
actccaattggcgatggccctgtccttttaccagacaaccattacctgtcgacacaatctgtcctttcgaaagatcccaacgaaaagcgtgaccac
atggtccttcttgagtttgtaactgctgctgggattacacatggcatggatgagctctacaaataaggatctgaagcttgggcccgaacaaaaact
catctcagaagaggatctgaatagcgccgtcgaccatcatcatcatcatcattgagtttaaacggtctccagcttggctgttttggcggatgagag
aagattttcagcctgatacagattaaatcagaacgcagaagcggtctgataaaacagaatttgcctggcggcagtagcgcggtggtcccacctgac
cccatgccgaactcagaagtgaaacgccgtagcgccgatggtagtgtggggtctccccatgcgagagtagggaactgccaggcatcaaataaaacg
aaaggctcagtcgaaagactgggcctttcgttttatctgttgtttgtcggtgaactggatcgtcgaccggctgctaacaaagcccgaaaggaagct
gagttggctgctgccaccgctgagcaataactagcataaccccttggggcctctaaacgggtcttgaggggttttttg</td></tr>
<tr><th>pSwitchB_sfGFP</th><th>Plasmid encoding sfGFP under regulation of toehold switch B</th></tr>
<tr><td colspan="2">T7 Promoter-SwitchB-sfGFP-TrrnB-T7 Terminator

taatacgactcactataggGGGTGCGTCCCATATCTATCTCTAATTCGTAGTAATGTCGGACTTTAGAACAGAGGAGATAAAGATGGACATTACTA
CAAACCTGGCGGCAGCGCAAAAGatgcgtaaaggagaagaacttttcactggagttgtcccaattcttgttgaattagatggtgatgttaatgggc
acaaattttctgtccgtggagagggtgaaggtgatgctacaaacggaaaactcacccttaaatttatttgcactactggaaaactacctgttccgt
ggccaacacttgtcactactctgacctatggtgttcaatgcttttcccgttatccggatcacatgaaacggcatgactttttcaagagtgccatgc
ccgaaggttatgtacaggaacgcactatatctttcaaagatgacgggacctacaagacgcgtgctgaagtcaagtttgaaggtgatacccttgtta
atcgtatcgagttaaagggtattgattttaaagaagatggaaacattcttggacacaaactcgagtacaactttaactcacacaatgtatacatca
cggcagacaaacaaaagaatggaatcaaagctaacttcaaaattcgccacaacgttgaagatggttccgttcaactagcagaccattatcaacaaa
atactccaattggcgatggccctgtccttttaccagacaaccattacctgtcgacacaatctgtcctttcgaaagatcccaacgaaaagcgtgacc
acatggtccttcttgagtttgtaactgctgctgggattacacatggcatggatgagctctacaaataaggatctgaagcttgggcccgaacaaaaa
ctcatctcagaagaggatctgaatagcgccgtcgaccatcatcatcatcatcattgagtttaaacggtctccagcttggctgttttggcggatgag
agaagattttcagcctgatacagattaaatcagaacgcagaagcggtctgataaaacagaatttgcctggcggcagtagcgcggtggtcccacctg
accccatgccgaactcagaagtgaaacgccgtagcgccgatggtagtgtggggtctccccatgcgagagtagggaactgccaggcatcaaataaaa</td></tr>
</table>

cgaaaggctcagtcgaaagactgggcctttcgttttatctgttgtttgtcggtgaactggatcgtcgaccggctgctaacaaagcccgaaaggaag
ctgagttggctgctgccaccgctgagcaataactagcataaccccttggggcctctaaacgggtcttgaggggttttttg

pSwitchC_sfGFP	Plasmid encoding sfGFP under regulation of toehold switch C

T7 Promoter-SwitchC-sfGFP-TrrnB-T7 Terminator

taatacgactcactatagggGGATACTATTTAACGAAGAGACATAACTTTGTTGGGTCGGACTTTAGAACAGAGGAGATAAAGATGGACCCAACAA
AGAACCTGGCGGCAGCGCAAAAGatgcgtaaaggagaagaacttttcactggagttgtcccaattcttgttgaattagatggtgatgttaatgggc
acaaattttctgtccgtggagagggtgaaggtgatgctacaaacggaaaactcacccttaaatttatttgcactactggaaaactacctgttccgt
ggccaacacttgtcactactctgacctatggtgttcaatgcttttcccgttatccggatcacatgaaacggcatgactttttcaagagtgccatgc
ccgaaggttatgtacaggaacgcactatatctttcaaagatgacgggacctacaagacgcgtgctgaagtcaagtttgaaggtgatacccttgtta
atcgtatcgagttaaagggtattgattttaaagaagatggaaacattcttggacacaaactcgagtacaactttaactcacacaatgtatacatca
cggcagacaaacaaaagaatggaatcaaagctaacttcaaaattcgccacaacgttgaagatggttccgttcaactagcagaccattatcaacaa
atactccaattggcgatggccctgtccttttaccagacaaccattacctgtcgacacaatctgtcctttcgaaagatcccaacgaaaagcgtgacc
acatggtccttcttgagtttgtaactgctgctgggattacacatggcatggatgagctctacaaataaggatctgaagcttgggcccgaacaaaaa
ctcatctcagaagaggatctgaatagcgccgtcgaccatcatcatcatcatcattgagtttaaacggtctccagcttggctgttttggcggatgag
agaagattttcagcctgatacagattaaatcagaacgcagaagcggtctgataaaacagaatttgcctggcggcagtagcgcggtggtcccacctg
accccatgccgaactcagaagtgaaacgccgtagcgccgatggtagtgtggggtctccccatgcgagagtagggaactgccaggcatcaaataaaa
cgaaaggctcagtcgaaagactgggcctttcgttttatctgttgtttgtcggtgaactggatcgtcgaccggctgctaacaaagcccgaaaggaag
ctgagttggctgctgccaccgctgagcaataactagcataaccccttggggcctctaaacgggtcttgaggggttttttg

pSwitchD_sfGFP	Plasmid encoding sfGFP under regulation of toehold switch D

T7 Promoter-SwitchD-sfGFP-TrrnB-T7 Terminator

taatacgactcactatagggGGCCTACTAAATTCAACTACGCAATCCGTGTACAAGCCGGACTTTAGAACAGAGGAGATAAAGATGGCTTGTACA
CAAACCTGGCGGCAGCGCAAAAGatgcgtaaaggagaagaacttttcactggagttgtcccaattcttgttgaattagatggtgatgttaatgggc
acaaattttctgtccgtggagagggtgaaggtgatgctacaaacggaaaactcacccttaaatttatttgcactactggaaaactacctgttccgt
ggccaacacttgtcactactctgacctatggtgttcaatgcttttcccgttatccggatcacatgaaacggcatgactttttcaagagtgccatgc
ccgaaggttatgtacaggaacgcactatatctttcaaagatgacgggacctacaagacgcgtgctgaagtcaagtttgaaggtgatacccttgtta
atcgtatcgagttaaagggtattgattttaaagaagatggaaacattcttggacacaaactcgagtacaactttaactcacacaatgtatacatca
cggcagacaaacaaaagaatggaatcaaagctaacttcaaaattcgccacaacgttgaagatggttccgttcaactagcagaccattatcaacaa
atactccaattggcgatggccctgtccttttaccagacaaccattacctgtcgacacaatctgtcctttcgaaagatcccaacgaaaagcgtgacc
acatggtccttcttgagtttgtaactgctgctgggattacacatggcatggatgagctctacaaataaggatctgaagcttgggcccgaacaaaaa
ctcatctcagaagaggatctgaatagcgccgtcgaccatcatcatcatcatcattgagtttaaacggtctccagcttggctgttttggcggatgag
agaagattttcagcctgatacagattaaatcagaacgcagaagcggtctgataaaacagaatttgcctggcggcagtagcgcggtggtcccacctg
accccatgccgaactcagaagtgaaacgccgtagcgccgatggtagtgtggggtctccccatgcgagagtagggaactgccaggcatcaaataaaa

cgaaaggctcagtcgaaagactgggcctttcgttttatctgttgtttgtcggtgaactggatcgtcgaccggctgctaacaaagcccgaaaggaag
ctgagttggctgctgccaccgctgagcaataactagcataaccccttggggcctctaaacgggtcttgaggggttttttg

p3WJ_N20Switch_sfGFP	Plasmid encoding sfGFP under regulation of the 3WJ N20 switch

T7 Promoter-3WJRepresssor N20-sfGFP-TrrnB-T7 Terminator

taatacgactcactataggGGGatgaatgatatacacttgttatagttatgaacagaggagacataacatgaacaagcacgaattgactacactAA
ACCTGGCGGCAGCGCAAAAGatgcgtaaaggagaagaacttttcactggagttgtcccaattcttgttgaattagatggtgatgttaatgggcaca
aattttctgtccgtggagagggtgaaggtgatgctacaaacggaaaactcacccttaaatttatttgcactactggaaaactacctgttccgtggc
caacacttgtcactactctgacctatggtgttcaatgcttttcccgttatccggatcacatgaaacggcatgactttttcaagagtgccatgcccg
aaggttatgtacaggaacgcactatatctttcaaagatgacgggacctacaagacgcgtgctgaagtcaagtttgaaggtgatacccttgttaatc
gtatcgagttaaagggtattgattttaaagaagatggaaacattcttggacacaaactcgagtacaactttaactcacacaatgtatacatcacgg
cagacaaacaaaagaatggaatcaaagctaacttcaaaattcgccacaacgttgaagatggttccgttcaactagcagaccattatcaacaaaata
ctccaattggcgatggccctgtccttttaccagacaaccattacctgtcgacacaatctgtcctttcgaaagatcccaacgaaaagcgtgaccaca
tggtccttcttgagtttgtaactgctgctgggattacacatggcatggatgagctctacaaataaggatctgaagcttgggcccgaacaaaaactc
atctcagaagaggatctgaatagcgccgtcgaccatcatcatcatcatcattgagtttaaacggtctccagcttggctgttttggcggatgagaga
agattttcagcctgatacagattaaatcagaacgcagaagcggtctgataaaacagaatttgcctggcggcagtagcgcggtggtcccacctgacc
ccatgccgaactcagaagtgaaacgccgtagcgccgatggtagtgtggggtctccccatgcgagagtagggaactgccaggcatcaaataaaacga
aaggctcagtcgaaagactgggcctttcgttttatctgttgtttgtcggtgaactggatcgtcgaccggctgctaacaaagcccgaaaggaagctg
agttggctgctgccaccgctgagcaataactagcataaccccttggggcctctaaacgggtcttgaggggttttttg

pTriggerATemp	Plasmid with TriggerA consensus sequence and terminator

TriggerA Consensus-T7 Terminator

AAGGAGCAACACCACACAAAGCCtagcataaccccttggggcctctaaacgggtcttgaggggttttttg

pTriggerBTemp	Plasmid with TriggerB consensus sequence and terminator

TriggerB Consensus-T7 Terminator

GACATTACTACGAATTAGAGATAtagcataaccccttggggcctctaaacgggtcttgaggggttttttg

pTriggerCTemp	Plasmid with TriggerC consensus sequence and terminator

TriggerC Consensus-T7 Terminator

CGACCCAACAAAGTTATGTCTCtagcataaccccttggggcctctaaacgggtcttgaggggttttttg

**pTriggerDTemp**	**Plasmid with TriggerD consensus sequence and terminator**
TriggerD Consensus-T7 Terminator<br><br>GGCTTGTACACGGATTGCGTAGtagcataaccccttggggcctctaaacgggtcttgaggggttttttg	
**p3WJ_N20RepressTemp**	**Plasmid with 3WJ N20 consensus sequence and terminator**
3WJ N20 Consensus-T7 Terminator<br><br>tagtgtagtcaattcgtgtagcataaccccttggggcctctaaacgggtcttgaggggttttttg	

Table S3: Primer sequences used in this study

All triggers used in this study were expressed from linear DNA expression templates. These templates were synthesized via PCR with the primers described in the tables below. PCR template plasmids for each trigger amplification are described in Table S2. Each forward primer contains the T7 promoter (lowercase) on the 5' end such that the amplicon can be used to transcribe the encoded RNA trigger in the CFE reaction. In some cases, additional downstream nucleotides (lowercase) were included in the priming region to achieve suitable annealing temperatures. Red nucleotides indicate single nucleotide mutations in the consensus trigger sequence.

Table S3A: Primer sequences used for TriggerA syntheses

**Trigger Name**	**Encoded Trigger Sequence (5′ → 3′)**	**Forward Primer (5′ → 3′)**	**Figure(s)**
TA001	AAGGAGCAACACCACACAAAGCC	CtaatacgactcactataggAAGGAGCAACACCACACAAAGC	1, S5, 3, S6
TA002	CAGGAGCAACACCACACAAAGCC	CtaatacgactcactataggCAGGAGCAACACCACACAAAGC	1B
TA003	ACGGAGCAACACCACACAAAGCC	CtaatacgactcactataggACGGAGCAACACCACACAAAG	1B, 1C
TA067	AAUGAGCAACACCACACAAAGCC	CtaatacgactcactataggAATGAGCAACACCACACAAAGC	1B
TA053	AAGUAGCAACACCACACAAAGCC	CtaatacgactcactataggAAGTAGCAACACCACACAAAGCC	1B, 1C
TA006	AAGGCGCAACACCACACAAAGCC	CtaatacgactcactataggAAGGCGCAACACCACACAAAG	1B
TA069	AAGGAUCAACACCACACAAAGCC	CtaatacgactcactataggAAGGATCAACACCACACAAAGCC	1B
TA008	AAGGAGAAACACCACACAAAGCC	CtaatacgactcactataggAAGGAGAAACACCACACAAAGCC	1B
TA009	AAGGAGCCACACCACACAAAGCC	CtaatacgactcactataggAAGGAGCCACACCACACAAAG	1B, 1C
TA010	AAGGAGCACCACCACACAAAGCC	CtaatacgactcactataggAAGGAGCACCACCACACAAAG	1B
TA011	AAGGAGCAAAACCACACAAAGCC	CtaatacgactcactataggAAGGAGCAAAACCACACAAAGCC	1B, 1C
TA012	AAGGAGCAACCCCACACAAAGCC	CtaatacgactcactataggAAGGAGCAACCCCACACAAAG	1B
TA013	AAGGAGCAACAACACACAAAGCC	CtaatacgactcactataggAAGGAGCAACAACACACAAAGCC	1B
TA014	AAGGAGCAACACAACACAAAGCC	CtaatacgactcactataggAAGGAGCAACACAACACAAAGCC	1B
TA015	AAGGAGCAACACCCCACAAAGCC	CtaatacgactcactataggAAGGAGCAACACCCCACAAAG	1B, 1C
TA016	AAGGAGCAACACCAAACAAAGCC	CtaatacgactcactataggAAGGAGCAACACCAAACAAAGCC	1B
TA017	AAGGAGCAACACCACCCAAAGCC	CtaatacgactcactataggAAGGAGCAACACCACCCAAAG	1B, 1C
TA018	AAGGAGCAACACCACAAAAAGCC	CtaatacgactcactataggAAGGAGCAACACCACAAAAAGCC	1B
TA019	AAGGAGCAACACCACACCAAGCC	CtaatacgactcactataggAAGGAGCAACACCACACCAAG	1B
TA020	AAGGAGCAACACCACACACAGCC	CtaatacgactcactataggAAGGAGCAACACCACACACAG	1B
TA021	AAGGAGCAACACCACACAACGCC	CtaatacgactcactataggAAGGAGCAACACCACACAACG	1B, 1C
TA071	AAGGAGCAACACCACACAAAUCC	CtaatacgactcactataggAAGGAGCAACACCACACAAATCC	1B
TA023	AAGGAGCAACACCACACAAAGAC	CtaatacgactcactataggAAGGAGCAACACCACACAAAGAC	1B, 1C
TA024	AAGGAGCAACACCACACAAAGCA	CtaatacgactcactataggAAGGAGCAACACCACACAAAGCA	1B

TA073	AAGGAGUAACACCACACAAAGCC	CtaatacgactcactataggAAGGAGTAACACCACACAAAGCC	1D
TA074	AAGGAGCAAUACCACACAAAGCC	CtaatacgactcactataggAAGGAGCAATACCACACAAAGCC	1D
TA075	AAGGAGCAACAUCACACAAAGCC	CtaatacgactcactataggAAGGAGCAACATCACACAAAGCC	1D
TA076	AAGGAGCAACACUACACAAAGCC	CtaatacgactcactataggAAGGAGCAACACTACACAAAGCC	1D
TA077	AAGGAGCAACACCAUACAAAGCC	CtaatacgactcactataggAAGGAGCAACACCATACAAAGCC	1D
TA078	AAGGAGCAACACCACAUAAAGCC	CtaatacgactcactataggAAGGAGCAACACCACATAAAGCC	1D
TA079	AAGGAGCAACACCACACAAAGUC	CtaatacgactcactataggAAGGAGCAACACCACACAAAGTC	1D
TA080	AAGGAGCAACACCACACAAAGCU	CtaatacgactcactataggAAGGAGCAACACCACACAAAGCT	1D
TA081	AAGCGAGCAACACCACACAAAGCC	CtaatacgactcactataggAAGCGAGCAACACCACACAAA	1E
TA082	AAGGAGCACACACCACACAAAGCC	CtaatacgactcactataggAAGGAGCACACACCACACAAAG	1E
TA083	AAGGAGCAACACCACCACAAAGCC	CtaatacgactcactataggAAGGAGCAACACCACCACAAAG	1E
TA084	AAGGAGCAACACCACACAACAGCC	CtaatacgactcactataggAAGGAGCAACACCACACAACAG	1E
TA085	AAGAGCAACACCACACAAAGCC	CtaatacgactcactataggAAGAGCAACACCACACAAAGC	1E
TA086	AAGGAGCACACCACACAAAGCC	CtaatacgactcactataggAAGGAGCACACCACACAAAGC	1E
TA087	AAGGAGCAACACCAACAAAGCC	CtaatacgactcactataggAAGGAGCAACACCAACAAAGCC	1E
TA088	AAGGAGCAACACCACACAAGCC	CtaatacgactcactataggAAGGAGCAACACCACACAAGC	1E
TA095	UAGGAGCAACACCACACAAAGCC	CtaatacgactcactataggTAGGAGCAACACCACACAAAGC	S5A
TA096	AUGGAGCAACACCACACAAAGCC	CtaatacgactcactataggATGGAGCAACACCACACAAAGC	S5A
TA097	AAGGUGCAACACCACACAAAGCC	CtaatacgactcactataggAAGGTGCAACACCACACAAAGC	S5A
TA098	AAGGAGCUACACCACACAAAGCC	CtaatacgactcactataggAAGGAGCTACACCACACAAAGC	S5A
TA099	AAGGAGCAUCACCACACAAAGCC	CtaatacgactcactataggAAGGAGCATCACCACACAAAGC	S5A
TA100	AAGGAGCAACUCCACACAAAGCC	CtaatacgactcactataggAAGGAGCAACTCCACACAAAGC	S5A
TA101	AAGGAGCAACACCUCACAAAGCC	CtaatacgactcactataggAAGGAGCAACACCTCACAAAGC	S5A
TA102	AAGGAGCAACACCACUCAAAGCC	CtaatacgactcactataggAAGGAGCAACACCACTCAAAGC	S5A
TA103	AAGGAGCAACACCACACUAAGCC	CtaatacgactcactataggAAGGAGCAACACCACACTAAGCC	S5A
TA104	AAGGAGCAACACCACACAUAGCC	CtaatacgactcactataggAAGGAGCAACACCACACATAGCC	S5A
TA105	AAGGAGCAACACCACACAAUGCC	CtaatacgactcactataggAAGGAGCAACACCACACAATGCC	S5A
TA055	AAGGAGCAACACGACACAAAGCC	CtaatacgactcactataggAAGGAGCAACACGACACAAAGC	S5B
TA057	AAGGAGGAACACCACACAAAGCC	CtaatacgactcactataggAAGGAGGAACACCACACAAAGC	S5B
TA058	AAGGAGCAAGACCACACAAAGCC	CtaatacgactcactataggAAGGAGCAAGACCACACAAAGC	S5B
TA059	AAGGAGCAACAGCACACAAAGCC	CtaatacgactcactataggAAGGAGCAACAGCACACAAAGC	S5B
TA060	AAGGAGCAACACCAGACAAAGCC	CtaatacgactcactataggAAGGAGCAACACCAGACAAAGC	S5B
TA061	AAGGAGCAACACCACAGAAAGCC	CtaatacgactcactataggAAGGAGCAACACCACAGAAAGC	S5B
TA062	AAGGAGCAACACCACACAAAGGC	CtaatacgactcactataggAAGGAGCAACACCACACAAAGGC	S5B
TA063	AAGGAGCAACACCACACAAAGCG	CtaatacgactcactataggAAGGAGCAACACCACACAAAGCG	S5B
TA068	AACGAGCAACACCACACAAAGCC	CtaatacgactcactataggAACGAGCAACACCACACAAAG	S5C

TA004	AAAGAGCAACACCACACAAAGCC	CtaatacgactcactataggAAAGAGCAACACCACACAAAGCC	S5C
TA054	AAGCAGCAACACCACACAAAGCC	CtaatacgactcactataggAAGCAGCAACACCACACAAAGC	S5C
TA005	AAGAAGCAACACCACACAAAGCC	CtaatacgactcactataggAAGAAGCAACACCACACAAAGCC	S5C
TA070	AAGGACCAACACCACACAAAGCC	CtaatacgactcactataggAAGGACCAACACCACACAAAGC	S5C
TA007	AAGGAACAACACCACACAAAGCC	CtaatacgactcactataggAAGGAACAACACCACACAAAGCC	S5C
TA072	AAGGAGCAACACCACACAAACCC	CtaatacgactcactataggAAGGAGCAACACCACACAAACC	S5C
TA022	AAGGAGCAACACCACACAAAACC	CtaatacgactcactataggAAGGAGCAACACCACACAAAACC	S5C
TA032	AAGGAGAAACAACACACAAAGCC	CtaatacgactcactataggAAGGAGAAACAACACACAAAGCC	3
TA038	AAGGCGCAAAACCACACAAAGCC	CtaatacgactcactataggAAGGCGCAAAACCACACAAAGC	3
TA041	AAGGAGCAACACCCCACAAAGAC	CtaatacgactcactataggAAGGAGCAACACCCCACAAAGAC	3
TA042	AAGGAGCAACACCACACAAAGAA	CtaatacgactcactataggAAGGAGCAACACCACACAAAGAA	3
TA044	AAGAAGCAAAACCACCCAAAGCC	CtaatacgactcactataggAAGAAGCAAAACCACCCAAAGCC	3
TA045	AAGGAGCCACACCCCACAACGCC	CtaatacgactcactataggAAGGAGCCACACCCCACAAC	3
TA046	CAGGAGCCACACCAAACAAAGCA	CtaatacgactcactataggCAGGAGCCACACCAAACAAAGCA	3
TA047	CAGGAGAACCCCCACACAAAGCC	CtaatacgactcactataggCAGGAGAACCCCCACACAAAGC	3
TA048	AAGGAGCAACACAACCCACAGAC	CtaatacgactcactataggAAGGAGCAACACAACCCACAGAC	3
TA049	ACGGAACAACCCCACAAAAAACC	CtaatacgactcactataggACGGAACAACCCCACAAAAAACC	3
TA050	CAAGCGCCACAACACACAAAGCC	CtaatacgactcactataggCAAGCGCCACAACACACAAAGC	3
TA051	AAGGAGCAACACCCAACACAACA	CtaatacgactcactataggAAGGAGCAACACCCAACACAACA	3
TA052	CAGGAGCACCAACAAACACAGAC	CtaatacgactcactataggCAGGAGCACCAACAAACACAGAC	3
TA064	AAGGAGCAACACCCAAACAAAAC	CtaatacgactcactataggAAGGAGCAACACCCAAACAAAACtagcat	3
TA065	AAGGAGCAACACAAACAACAAAC	CtaatacgactcactataggAAGGAGCAACACAAACAACAAACtagcat	3
TA066	AAGGAGCAACACACCCAACCGAA	CtaatacgactcactataggAAGGAGCAACACACCCAACCGAAtagcat	3
TA089	AAGGAGCAACACCAAAACAAAAC	CtaatacgactcactataggAAGGAGCAACACCAAAACAAAACtag	S6
TA090	AAGGAGCAACACCCCAACAAAAC	CtaatacgactcactataggAAGGAGCAACACCCCAACAAAAC	S6
TA091	AAGGAGCAACACCCAACCAAAAC	CtaatacgactcactataggAAGGAGCAACACCCAACCAAAAC	S6
TA092	AAGGAGCAACACCCAAAAAAAAC	CtaatacgactcactataggAAGGAGCAACACCCAAAAAAAACTAG	S6
TA093	AAGGAGCAACACCCAAACAAGAC	CtaatacgactcactataggAAGGAGCAACACCCAAACAAGAC	S6
TA094	AAGGAGCAACACCCAAACAAACC	CtaatacgactcactataggAAGGAGCAACACCCAAACAAACC	S6

Table S3B: Primer sequences used for TriggerB syntheses

TB001	GACAUUACUACGAAUUAGAGAUA	CtaatacgactcactataggGACATTACTACGAATTAGAGATAtagc	2, S2, S3, S3, S4, S7A
TB002	UACAUUACUACGAAUUAGAGAUA	CtaatacgactcactataggTACATTACTACGAATTAGAGATAtagc	S2
TB003	GCCAUUACUACGAAUUAGAGAUA	CtaatacgactcactataggGCCATTACTACGAATTAGAGATAtagc	S2
TB004	GAAAUUACUACGAAUUAGAGAUA	CtaatacgactcactataggGAAATTACTACGAATTAGAGATAtagc	S2
TB005	GACCUUACUACGAAUUAGAGAUA	CtaatacgactcactataggGACCTTACTACGAATTAGAGATAtagc	S2
TB006	GACAGUACUACGAAUUAGAGAUA	CtaatacgactcactataggGACAGTACTACGAATTAGAGATAtagc	S2
TB007	GACAUGACUACGAAUUAGAGAUA	CtaatacgactcactataggGACATGACTACGAATTAGAGATAtagc	S2
TB008	GACAUUCCUACGAAUUAGAGAUA	CtaatacgactcactataggGACATTCCTACGAATTAGAGATAtagc	S2
TB009	GACAUUAAUACGAAUUAGAGAUA	CtaatacgactcactataggGACATTAATACGAATTAGAGATAtagc	S2
TB010	GACAUUACGACGAAUUAGAGAUA	CtaatacgactcactataggGACATTACGACGAATTAGAGATAtagc	S2
TB011	GACAUUACUCCGAAUUAGAGAUA	CtaatacgactcactataggGACATTACTCCGAATTAGAGATAtagc	S2
TB012	GACAUUACUAAGAAUUAGAGAUA	CtaatacgactcactataggGACATTACTAAGAATTAGAGATAtagc	S2
TB013	GACAUUACUACUAAUUAGAGAUA	CtaatacgactcactataggGACATTACTACTAATTAGAGATAtagc	S2
TB014	GACAUUACUACGCAUUAGAGAUA	CtaatacgactcactataggGACATTACTACGCATTAGAGATAtagc	S2
TB015	GACAUUACUACGACUUAGAGAUA	CtaatacgactcactataggGACATTACTACGACTTAGAGATAtagc	S2
TB016	GACAUUACUACGAAGUAGAGAUA	CtaatacgactcactataggGACATTACTACGAAGTAGAGATAtagc	S2
TB017	GACAUUACUACGAAUGAGAGAUA	CtaatacgactcactataggGACATTACTACGAATGAGAGATAtagc	S2
TB018	GACAUUACUACGAAUUCGAGAUA	CtaatacgactcactataggGACATTACTACGAATTCGAGATAtagc	S2
TB019	GACAUUACUACGAAUUAUAGAUA	CtaatacgactcactataggGACATTACTACGAATTATAGATAtagc	S2
TB020	GACAUUACUACGAAUUAGCGAUA	CtaatacgactcactataggGACATTACTACGAATTAGCGATAtagc	S2
TB021	GACAUUACUACGAAUUAGAUAUA	CtaatacgactcactataggGACATTACTACGAATTAGATATAtagc	S2
TB022	GACAUUACUACGAAUUAGAGCUA	CtaatacgactcactataggGACATTACTACGAATTAGAGCTAtagc	S2
TB023	GACAUUACUACGAAUUAGAGAGA	CtaatacgactcactataggGACATTACTACGAATTAGAGAGAtagc	S2
TB024	GACAUUACUACGAAUUAGAGAUC	CtaatacgactcactataggGACATTACTACGAATTAGAGATCtagc	S2
TB060	GAUAUUACUACGAAUUAGAGAUA	CtaatacgactcactataggGATATTACTACGAATTAGAGATAtagc	S3
TB061	GACAUUAUUACGAAUUAGAGAUA	CtaatacgactcactataggGACATTATTACGAATTAGAGATAtagc	S3
TB062	GACAUUACUAUGAAUUAGAGAUA	CtaatacgactcactataggGACATTACTATGAATTAGAGATAtagc	S3
TB067	GACAUACUACGAAUUAGAGAUA	CtaatacgactcactataggGACATACTACGAATTAGAGATAtagca	S4B
TB068	GACAUUGACUACGAAUUAGAGAUA	CtaatacgactcactataggGACATTGACTACGAATTAGAGATAtagc	S4A
TB069	GACAUUCACUACGAAUUAGAGAUA	CtaatacgactcactataggGACATTCACTACGAATTAGAGATAtagc	S4A
TB070	GACAUUACUAGAAUUAGAGAUA	CtaatacgactcactataggGACATTACTAGAATTAGAGATAtagcat	S4B
TB071	GACAUUACUACUGAAUUAGAGAUA	CtaatacgactcactataggGACATTACTACTGAATTAGAGATAtagc	S4A
TB072	GACAUUACUACAGAAUUAGAGAUA	CtaatacgactcactataggGACATTACTACAGAATTAGAGATAtagc	S4A
TB073	GACAUUACUACGAAUUAGAAUA	CtaatacgactcactataggGACATTACTACGAATTAGAATAtagcat	S4B

TB074	GACAUUACUACGAAUUAGAGUAUA	CtaatacgactcactataggGACATTACTACGAATTAGAGTATAtagc	S4A
TB075	GACAUUACUACGAAUUAGAGCAUA	CtaatacgactcactataggGACATTACTACGAATTAGAGCATAtagc	S4A
TB076	GAGAUUACUACGAAUUAGAGAUA	CtaatacgactcactataggGAGATTACTACGAATTAGAGATAtagcat	2B
TB077	GACAUUAGUACGAAUUAGAGAUA	CtaatacgactcactataggGACATTAGTACGAATTAGAGATAtagcat	2B
TB078	GACAUUACUAGGAAUUAGAGAUA	CtaatacgactcactataggGACATTACTAGGAATTAGAGATAtagcat	2B
TB079	AACAUUACUACGAAUUAGAGAUA	CtaatacgactcactataggAACATTACTACGAATTAGAGATAtagcat	2C
TB080	CACAUUACUACGAAUUAGAGAUA	CtaatacgactcactataggCACATTACTACGAATTAGAGATAtagcat	2C
TB081	GACAUUACUACAAAUUAGAGAUA	CtaatacgactcactataggGACATTACTACAAATTAGAGATAtagcat	2C
TB082	GACAUUACUACCAAUUAGAGAUA	CtaatacgactcactataggGACATTACTACCAATTAGAGATAtagcat	2C
TB083	GACAUUACUACGAAUUAAAGAUA	CtaatacgactcactataggGACATTACTACGAATTAAAGATAtagcat	2C
TB084	GACAUUACUACGAAUUACAGAUA	CtaatacgactcactataggGACATTACTACGAATTACAGATAtagcat	2C
TB085	GACAUUACUACGAAUUAGAAAUA	CtaatacgactcactataggGACATTACTACGAATTAGAAATAtagcat	2C
TB086	GACAUUACUACGAAUUAGACAUA	CtaatacgactcactataggGACATTACTACGAATTAGACATAtagcat	2C
TB087	GUCAUUACUACGAAUUAGAGAUA	CtaatacgactcactataggGTCATTACTACGAATTAGAGATAtagcat	2A
TB088	GACUUUACUACGAAUUAGAGAUA	CtaatacgactcactataggGACTTTACTACGAATTAGAGATAtagcat	2A
TB089	GACAUUUCUACGAAUUAGAGAUA	CtaatacgactcactataggGACATTTCTACGAATTAGAGATAtagcat	2A
TB090	GACAUUACUUCGAAUUAGAGAUA	CtaatacgactcactataggGACATTACTTCGAATTAGAGATAtagcat	2A
TB091	GACAUUACUACGUAUUAGAGAUA	CtaatacgactcactataggGACATTACTACGTATTAGAGATAtagcat	2A
TB092	GACAUUACUACGAUUUAGAGAUA	CtaatacgactcactataggGACATTACTACGATTTAGAGATAtagcat	2A
TB093	GACAUUACUACGAAUUUGAGAUA	CtaatacgactcactataggGACATTACTACGAATTTGAGATAtagcat	2A
TB094	GACAUUACUACGAAUUAGUGAUA	CtaatacgactcactataggGACATTACTACGAATTAGTGATAtagcat	2A
TB095	GACAUUACUACGAAUUAGAGUUA	CtaatacgactcactataggGACATTACTACGAATTAGAGTTAtagcat	2A
TB096	GACAUUACUACGAAUUAGAGAUU	CtaatacgactcactataggGACATTACTACGAATTAGAGATTtagcat	2A
TB097	GACAAUACUACGAAUUAGAGAUA	CtaatacgactcactataggGACAATACTACGAATTAGAGATAtagc	2D
TB098	GACACUACUACGAAUUAGAGAUA	CtaatacgactcactataggGACACTACTACGAATTAGAGATAtagc	2D
TB099	GACAUAACUACGAAUUAGAGAUA	CtaatacgactcactataggGACATAACTACGAATTAGAGATAtagc	2D
TB100	GACAUCACUACGAAUUAGAGAUA	CtaatacgactcactataggGACATCACTACGAATTAGAGATAtagc	2D
TB101	GACAUUACAACGAAUUAGAGAUA	CtaatacgactcactataggGACATTACAACGAATTAGAGATAtagc	2D
TB102	GACAUUACCACGAAUUAGAGAUA	CtaatacgactcactataggGACATTACCACGAATTAGAGATAtagc	2D
TB103	GACAUUACUACGAAAUAGAGAUA	CtaatacgactcactataggGACATTACTACGAAATAGAGATAtagc	2D
TB104	GACAUUACUACGAACUAGAGAUA	CtaatacgactcactataggGACATTACTACGAACTAGAGATAtagc	2D
TB105	GACAUUACUACGAAUAAGAGAUA	CtaatacgactcactataggGACATTACTACGAATAAGAGATAtagc	2D
TB106	GACAUUACUACGAAUCAGAGAUA	CtaatacgactcactataggGACATTACTACGAATCAGAGATAtagc	2D
TB107	GACAUUACUACGAAUUAGAGAAA	CtaatacgactcactataggGACATTACTACGAATTAGAGAAAtagc	2D
TB108	GACAUUACUACGAAUUAGAGACA	CtaatacgactcactataggGACATTACTACGAATTAGAGACAtagc	2D

Table S3C: Primer sequences used for TriggerC syntheses

TC001	CGACCCAACAAAGUUAUGUCUC	CtaatacgactcactataggCGACCCAACAAAGTTATGTCTC	1C
TC002	CGAACCAACAAAGUUAUGUCUC	CtaatacgactcactataggCGAACCAACAAAGTTATGTCTC	1C
TC003	CGACCCAACCAAGUUAUGUCUC	CtaatacgactcactataggCGACCCAACCAAGTTATGTC	1C
TC004	CGACCCAACAAAGUCAUGUCUC	CtaatacgactcactataggCGACCCAACAAAGTCATGTC	1C
TC005	CGACCCAACAAAGUUAUGUCCC	CtaatacgactcactataggCGACCCAACAAAGTTATGTCC	1C

Table S3D: Primer sequences used for TriggerD syntheses

TD001	GGCUUGUACACGGAUUGCGUAG	CtaatacgactcactataggGGCTTGTACACGGATTGCG	1C, S7B
TD002	GACUUGUACACGGAUUGCGUAG	CtaatacgactcactataggGACTTGTACACGGATTGCGTAG	1C
TD003	GGCCUGUACACGGAUUGCGUAG	CtaatacgactcactataggGGCCTGTACACGGATTGC	1C
TD005	GGCUUGUCCACGGAUUGCGUAG	CtaatacgactcactataggGGCTTGTCCACGGATTGC	1C
TD006	GGCUUGUACCCGGAUUGCGUAG	CtaatacgactcactataggGGCTTGTACCCGGATTGC	1C
TD008	GGCUUGUACACGGCUUGCGUAG	CtaatacgactcactataggGGCTTGTACACGGCTTGC	1C
TD009	GGCUUGUACACGGAUCGCGUAG	CtaatacgactcactataggGGCTTGTACACGGATCGC	1C
TD010	GGCUUGUACACGGAUUGAGUAG	CtaatacgactcactataggGGCTTGTACACGGATTGAGTAG	1C
TD011	GGCUUGUACACGGAUUGCGCAG	CtaatacgactcactataggGGCTTGTACACGGATTGCGC	1C
TD013	GGAUUGUCCACGGAUUGCGUAG	CtaatacgactcactataggGGATTGTCCACGGATTGCGTAG	S7B
TD015	GGCUUGUACACGGACUGCGUCG	CtaatacgactcactataggGGCTTGTACACGGACTGCGTC	S7B
TD016	GACUUAUACCCGGAUUGCGUAG	CtaatacgactcactataggGACTTATACCCGGATTGCGTAGt	S7B
TD018	GGCUUGUACACGAAUUACGUCG	CtaatacgactcactataggGGCTTGTACACGAATTACGTCGt	S7B
TD019	AGCCUGCACCCGGAUUGCGUAG	CtaatacgactcactataggAGCCTGCACCCGGATTGC	S7B
TD021	GGCUUGUACACAGACUGAGUCG	CtaatacgactcactataggGGCTTGTACACAGACTGAGTCG	S7B
TD022	GGCUUGUACACAGCUUACAUCG	CtaatacgactcactataggGGCTTGTACACAGCTTACATCGtagc	S7B

Table S3E: Primer sequences used for Repressor syntheses

R001	UAGUGUAGUCAAUUCGUG	CtaatacgactcactataggTAGTGTAGTCAATTCGTGtagc	4B, 4C
R002	GAGUGUAGUCAAUUCGUG	CtaatacgactcactataggGAGTGTAGTCAATTCGTGtagc	4C
R003	UCGUGUAGUCAAUUCGUG	CtaatacgactcactataggTCGTGTAGTCAATTCGTGtag	4C
R004	UAUUGUAGUCAAUUCGUG	CtaatacgactcactataggTATTGTAGTCAATTCGTGtagcat	4C
R005	UAGGGUAGUCAAUUCGUG	CtaatacgactcactataggTAGGGTAGTCAATTCGTGtagc	4C
R006	UAGUUUAGUCAAUUCGUG	CtaatacgactcactataggTAGTTTAGTCAATTCGTGtagcat	4C
R007	UAGUGGAGUCAAUUCGUG	CtaatacgactcactataggTAGTGGAGTCAATTCGTGtag	4C
R008	UAGUGUCGUCAAUUCGUG	CtaatacgactcactataggTAGTGTCGTCAATTCGTGtag	4C

R009	UAGUGUAUUCAAUUCGUG	CtaatacgactcactataggTAGTGTATTCAATTCGTGtagcat	4C
R010	UAGUGUAGGCAAUUCGUG	CtaatacgactcactataggTAGTGTAGGCAATTCGTGtag	4C
R011	UAGUGUAGUAAAUUCGUG	CtaatacgactcactataggTAGTGTAGTAAATTCGTGtagcataa	4C
R012	UAGUGUAGUCCAUUCGUG	CtaatacgactcactataggTAGTGTAGTCCATTCGTGtagc	4C
R013	UAGUGUAGUCACUUCGUG	CtaatacgactcactataggTAGTGTAGTCACTTCGTGtagc	4C
R014	UAGUGUAGUCAAGUCGUG	CtaatacgactcactataggTAGTGTAGTCAAGTCGTGtagc	4C
R015	UAGUGUAGUCAAUGCGUG	CtaatacgactcactataggTAGTGTAGTCAATGCGTGtag	4C
R016	UAGUGUAGUCAAUUAGUG	CtaatacgactcactataggTAGTGTAGTCAATTAGTGtagcataac	4C
R017	UAGUGUAGUCAAUUCUUG	CtaatacgactcactataggTAGTGTAGTCAATTCTTGtagcat	4C
R018	UAGUGUAGUCAAUUCGGG	CtaatacgactcactataggTAGTGTAGTCAATTCGGGtagc	4C
R019	UAGUGUAGUCAAUUCGUU	CtaatacgactcactataggTAGTGTAGTCAATTCGTTtagcat	4B
R020	UAGUGUAGUCAAUUCGUGC	CtaatacgactcactataggTAGTGTAGTCAATTCGTGCtagcat	4B
R021	UAGUGUAGUCAAUUCGUGCU	CtaatacgactcactataggTAGTGTAGTCAATTCGTGCTtagcat	4B
R022	UAGUGUAGUCAAUUCGUGCUU	CtaatacgactcactataggTAGTGTAGTCAATTCGTGCTTtagcat	4B
R023	UAGUGUAGUCAAUUCGUGCUUG	CtaatacgactcactataggTAGTGTAGTCAATTCGTGCTTGtagcat	4B

Table S3F: Reverse primers used for amplification

**Primer Name**	**Primer Sequence (5′ → 3′)**	**Templates used for**
68C.R	GCTTTTGCCATTCTCACCGGATT	SwitchA
62C.R	GCTTTTGCCATTCTCACC	SwitchB,C,D, and Repressor

References:

(1) Sun, Z. Z.; Hayes, C. A.; Shin, J.; Caschera, F.; Murray, R. M.; Noireaux, V. Protocols for Implementing an Escherichia Coli Based TX-TL Cell-Free Expression System for Synthetic Biology. *J. Vis. Exp. JoVE* **2013**, No. 79, e50762. https://doi.org/10.3791/50762.

(2) Green, A. A.; Silver, P. A.; Collins, J. J.; Yin, P. Toehold Switches: De-Novo-Designed Regulators of Gene Expression. *Cell* **2014**, *159* (4), 925–939. https://doi.org/10.1016/j.cell.2014.10.002.

(3) Pardee, K.; Green, A. A.; Takahashi, M. K.; Braff, D.; Lambert, G.; Lee, J. W.; Ferrante, T.; Ma, D.; Donghia, N.; Fan, M.; Daringer, N. M.; Bosch, I.; Dudley, D. M.; O'Connor, D. H.; Gehrke, L.; Collins, J. J. Rapid, Low-Cost Detection of Zika Virus Using Programmable Biomolecular Components. *Cell* **2016**, *165* (5), 1255–1266. https://doi.org/10.1016/j.cell.2016.04.059.
